# Enhancing the structural and optoelectronic properties of carboxymethyl cellulose sodium filled with ZnO/GO and CuO/GO nanocomposites for antimicrobial packaging applications

**DOI:** 10.1038/s41598-024-81365-3

**Published:** 2024-12-23

**Authors:** Rania Badry, Noha M. Sabry, Medhat A. Ibrahim

**Affiliations:** 1 Physics Department, Faculty of Women for Arts, Science and Education, Ain Shams University, Cairo, 11757 Egypt; 2 Water Pollution Research Department, Environment and Climate Change Research Institute, National Research Centre, 33 El-Bohouth St., Dokki, Giza, 12622 Egypt; 3 Center of Excellence for Research and Applied Studies on Climate Change and Sustainable Development, National Research Centre (NRC), 33 El Bohouth St., Dokki, Giza, 12622 Egypt; 4 Spectroscopy Department, National Research Centre, 33 El-Bohouth St., Dokki, Giza, 12622 Egypt; 5 Molecular Modeling and Spectroscopy Laboratory, Centre of Excellence for Advanced Science, National Research Centre, 33 El-Bohouth St., Dokki, Giza, 12622 Egypt

**Keywords:** UV shielding, CMC/ZnO or CuO/GO nanocomposites, FTIR, DFT, Antibacterial activity, Environmental sciences, Materials science

## Abstract

One of the biggest challenges in food packaging is the creation of sustainable and eco-friendly packaging materials to shield foods from ultraviolet (UV) photochemical damage and to preserve the distinctive physical, chemical, and biological characteristics of foods throughout the supply chain. Accordingly, this study focuses on enhancing the UV shielding properties and biological activity of carboxylmethyl cellulose sodium (CMC) through modifications using zinc oxide (ZnO), copper oxide (CuO), and graphene oxide (GO) using the solution casting technique. The hybrid nanocomposites were characterized by fourier-transform infrared (FTIR) spectrophotometer, ultraviolet-visible (UV-Vis) spectrophotometer, scanning electron microscopy (SEM), energy-dispersive X-ray spectroscopy (EDX), and x-ray diffraction (XRD). Significant interactions between CMC and the metal oxide/GO nanocomposites were revealed by FTIR analysis, which reflects the formation of hydrogen bonding between CMC and the nanocomposites. XRD confirmed the functionalization of CMC with ZnO/GO and CuO/GO nanocomposites. Additionally, the CMC film showed a decrease in the optical bandgap from 5.53 to 3.43 eV with improved UV shielding capacity. Moreover, the composite films had excellent refractive index and optical conductivity values of 1.97 and 1.56 × 10^10^ Ω cm^− 1^, respectively. SEM and EDX analysis confirmed the formation of ZnO/GO and CuO/GO within the CMC matrix. Thus, dedicates that the CMC nanocomposites have promising applications in packaging materials. These results were confirmed by the quantum mechanical calculations utilizing density functional theory (DFT). Total dipole moment (TDM), frontier molecular orbitals (FMOs), chemical reactivity descriptors, and molecular electrostatic potential (MESP) maps were all studied using the B3LYP/LanL2DZ model. The TDM and FMO investigations revealed that the CMC/CuO/GO model has the highest TDM (84.031 Debye) and the smallest band gap energy (0.118 eV). Moreover, CMC’s reactivity increased after CuO/GO nanocomposites integration, as demonstrated by MESP mapping. Finally, the antibacterial activity of pure CMC, CMC/ZnO/GO, and CMC/CuO/GO nanocomposite films was evaluated against *Staphylococcus aureus* and *Escherichia coli*. The zones of inhibition data showed that both CMC/ZnO/GO and CMC/CuO/GO exhibited higher antibacterial activity than CMC alone, particularly against *S. aureus*. The inhibition zones for CMC/ZnO/GO and CMC/CuO/GO against *S. aureus* were 16 mm and 14 mm, respectively, suggesting enhanced susceptibility of *S. aureus* compared to *E. coli*. These results highlight the significant potential of ZnO and CuO NPs in improving the antimicrobial efficacy of CMC nanocomposites.

## Introduction

Poorly biodegradable plastic packaging materials cause several kinds of environmental issues. As a result of severe environmental pollution and the depletion of nonrenewable resources, common synthetic plastic packaging is being replaced with renewable, biodegradable, and ecologically beneficial bio-based materials that have gained popularity in the packaging industry^1,2^. Thus, materials derived from biological sources that are biocompatible, renewable, and degradable have drawn a lot of interest in the packaging industry, especially in UV shielding applications^2^. Biodegradable substances comprise proteins (such as soy, whey, or corn protein), polysaccharides (such as cellulose, starch, bacterial cellulose, polyhydroxyalkylates, polylactic acid, and polyvinyl alcohol). From the materials previously mentioned, cellulose has proven to be an excellent material because of its easy water processing, good biodegradability, natural abundance, biocompatibility, and sustainability^3–5^. It is also useful for the preparation of multifunctional composites. Numerous industries, such as environmental remediation, aerospace, electronics, food preservation, antimicrobial materials, and wastewater treatment, use cellulose-based materials extensively^6–9^.

Despite having good transparency, cellulose films’ inability to block ultraviolet (UV) light prevents further development and application^10^. When polymer films are used to package food and biomedical goods, their ability to block UV light is crucial^11^. UV radiation plays a critical role in triggering DNA mutations and carcinogenesis^12^. In addition, UV radiation has the potential to seriously harm human skin and break down covalent bonds in organic polymers. Global research is focused on UV protective films and UV shielding coatings that block the entire UV spectrum while still allowing for good visible light transmission. The creation of sunscreen ingredients based on cellulose is crucial in this regard^13^.

To achieve UV protection, cellulose films are typically coated with organic UV absorbers (such as oxybenzone, avobenzone, and derivatives of benzophenone) and inorganic UV blockers (such as Ag particles, ZnO, TiO_2_, and ZnS) using a variety of intricate techniques^14^. For instance, benzophenone/PVA/carboxylated cellulose films were prepared by Niu et al.^15^, and the films showed good UV blocking performance. To block UV light, Youssef et al.^16^ integrated ZnO into composite films made of chitosan and CMC. On the other hand, the photocatalytic activity of inorganic absorbers and the self-degradation and permeation of organic absorbers restrict the use of each type of absorber.

CMC, derived from cellulose, a natural polymer found in plant cell walls, is a promising material in creating advanced and intelligent packaging materials^17^. This aligns with the packaging industry’s pursuit of sustainable alternatives. Cellulose, a primary structural component of plants, has inherent benefits like biocompatibility, renewability, and biodegradability^18^. Its natural hydrophobicity, however, prevents it from being used directly in many applications. This restriction is addressed by chemically adding carboxymethyl groups to the cellulose backbone, resulting in CMC, a water-soluble derivative with improved functionality^19^. The application of CMC in food packaging has been made possible by this modification process.

Regarding food packaging, CMC has demonstrated potential because of its film-forming characteristics, which include mechanical strength, transparency, barrier qualities, and thermal stability^20^. These film-forming materials exhibit good mechanical and barrier properties. CMC films typically have excellent transparency, high surface gloss, toughness, and tensile strength. In particular, CMC was found to have superior film-forming properties when compared to a water-soluble polymer and thermal gelatinization^21^.

Mechanical properties, such as tensile strength, rigidity, and elasticity, are among the most important ones, as they determine the material’s durability during storage, transportation, and handling. CMC is one of the biopolymers with the highest tensile strength and rigidity, but it may be less flexible. Plasticizers, such as glycerol, can be used to increase flexibility. Packaging that prolongs food’s shelf life can be created by incorporating antioxidant and antimicrobial compounds. Because of its positive charge, which interacts with bacteria’s negatively charged membranes to prevent their growth, CMC is well-known for its antimicrobial qualities^22^.

CMC is widely used in the food industry, dye absorption, and seawater desalination, among other applications^20–22^. It is also nontoxic and biocompatible. Among its many benefits are its low cost and good film-forming abilities. Microbial activity in the supply chain can cause food to deteriorate and rot, wasting valuable resources. Foodborne spoilage bacteria (e.g., *Streptococcus aureus and Escherichia coli*) can produce toxins that enter the human body and cause illness, affecting overall health. Antimicrobial packaging materials can be created by adding antimicrobial additives to cellulose-based materials that lack antimicrobial activity. This allows for wider market use^23^.

One commonly used technique for creating nanocomposite films is the solution casting technique. To attain the required dispersion and homogeneity in the prepared film-forming solution, the polymer composites and nanomaterials must be vigorously stirred or ultrasonically sonicated in the organic solvent or water. After that, the glass sheets or Petri plates can be coated with the film-forming solution. Depending on the components of the solution, the plates are left in a hot air oven to dry for at least 12–24 h. Thin films of composite materials made of a polymer matrix and nanomaterials are what remain after the solvent evaporates. The solution casting method has the benefit of not requiring specialized equipment for experimentation, relatively fast, low costs, uniform distribution of nanofiller, and, above all, simple changes in reaction conditions. But some drawbacks, like hydrophilicity and brittleness, have motivated scientists to investigate different strategies to improve the qualities of CMC-based films, making them appropriate for food packaging^24^.

To optimize CMC films in this context, numerous studies have been conducted on physical and chemical procedures, nano-scale filling substances, and blending with other polymers^25^. It has been demonstrated that adding inorganic (ZnO and CuO NPs) and organic nanoscale reinforcing agents (graphene oxide) can improve the mechanical characteristics of CMC films^26^. Depending on the concentration of the nanoparticle, metallic nanoparticles like ZnO and CuO have also been investigated for mechanical enhancement^27,28^.

Zinc oxide nanoparticles (ZnO NPs) are a common inorganic antibacterial material used in various fields, including medicine, pharmaceuticals, and wound dressing^29–31^. ZnO NPs have excellent antimicrobial properties and are biocompatible, making them ideal for incorporating into cellulose-based materials to create antimicrobial composites for food packaging applications^32^. When ZnO NPs are incorporated with carbon materials, various active sites of new materials facilitate the UV blocking and hence are used as antibacterial packaging material^33^.

Another crucial idea in food packaging is antimicrobial efficiency, which considers the regulation of the growth of microorganisms that cause food-borne illnesses. The development of microorganisms in the food packaging is not inhibited by bare plastic films. In the field of food packaging, ZnO and CuO NPs have attracted a lot of interest^16^. They present a number of benefits over traditional packaging materials, which make them a viable substitute for the future including large surface area and ability to generate reactive oxygen species (ROS), which contribute to bacterial membrane disruption and inhibition of metabolic processes^17^.

For the creation of UV shielding materials, graphene oxide (GO) sheets with strong photo-stability are appealing. Since conjugated aromatic structures are abundant in GO, they exhibit desirable UV shielding performance^34^. GO exhibits weak visible light absorption and strong UV zone absorption, as indicated by its optical band gap of 1.5 eV. GO has previously been incorporated into polymer matrices as UV absorbers and reinforcing fillers^35^. GO has a large surface area, is hydrophilic, and has a significant delocalized π-electron arrangement. According to Nebol’Sin et al.^36^, the abundance of oxygen atoms on the GO surface during the carboxy, hydroxyl, and epoxy functional groups helps combine with a variety of metal ions into its structure. Alves et al.^37^, used GO nanosheets in cellulose acetate to create UV shielding films. The composite film with 0.5 wt% GO loading had a UV shielding capacity of 57% and a visible light transparency of 79%.

Studies have demonstrated the effectiveness of ZnO and CuO NPs against a variety of pathogens, including *Staphylococcus aureus* and *Escherichia coli*^38,39^. The antibacterial action of these nanoparticles primarily involves ROS generation, which induces oxidative stress and leads to bacterial cell death^40^.

Accordingly, this work aims to improve the UV shielding property and the biological activity of CMC through the modification with ZnO/GO and CuO/GO nanocomposites and to advance computational methods in this area. The study involves the preparation of cast films at a constant metal oxide/GO concentration of 2 wt% and their subsequent analysis by FTIR and UV-Vis. The CMC/CuO/GO nanocomposites were modeled using density functional theory (DFT) calculations with the B3LYP/LanL2DZ model. DFT calculations were used to validate the proposed mechanism of interaction between CMC and GO nanocomposites and to trace the structural changes within the CMC matrix. The study goes on to assess TDM, HOMO/LUMO energy, and the chemical reactivity descriptors, offering insights with MESP maps. Also, the antibacterial activity of CMC/ZnO/GO and CMC/CuO/GO nanocomposite films was investigated against *S. aureus* and *E. coli*, aiming to understand how these nanocomposites enhance the antimicrobial properties of CMC.

## Materials and methods

### Materials

CMC with a molecular weight of 2.5 × 10^5^ g/mol was obtained from K. Patel Chemo-pharma PVT, India. Fisher Chemical supplied the sodium hydroxide (NaOH) (≥ 97%), potassium permanganate (KMnO4) (99%), phosphoric acid (85%), and graphite powder. Hydrogen peroxide (H_2_O_2_) (30%) was purchased from Piochem, and sulphuric acid (96%) was acquired from Scharlau. El Nasr Pharmaceutical Chemicals Co., Cairo, Egypt, supplied the absolute ethanol (analytical reagent, 99.9%) and sodium hydroxide (NaOH) pellets. Copper chloride (a 99% laboratory reagent) and zinc acetate dehydrate were obtained from Sigma-Aldrich in Germany. The glassware was thoroughly cleaned with soap solution before being washed with distilled water (D.W.). All the solutions were prepared with D.W.

### CuO NPs preparation

CuO NPs were prepared by following the same procedures as mentioned previously^12^. A magnetic stirrer was used to dissolve 0.2 M copper chloride in 100 mL of D.W. This solution was supplemented with 0.4 M NaOH, and the mixture was heated to approximately 50 °C for 4 h while maintaining a pH = 8. After a while, black precipitates will begin to accumulate at the beaker’s bottom. The particles were collected, centrifuged for 10 min at 8000 rpm, and washed three times in D.W. and once in ethanol. The collected residue was carefully transferred to an oven and heated to about 80 °C for drying (2 h) before being calcined at 500 °C for 2 h to produce CuO NPs.

### ZnO NPs preparation

ZnO NP was prepared by precipitation method by following the same procedures as mentioned previously^13^. At 70 °C, a 1 M zinc acetate dehydrate solution was made in 100 mL of D.W. 100 mL of D.W. contained a solution of 2 M NaOH. After vigorously stirring the zinc acetate solution for an hour, NaOH was added dropwise. Centrifugation at 1000 rpm and three rounds of D.W. washing was used to separate the white precipitate. The resultant precipitate was calcined for two hours at 500 °C and dried overnight in a dryer set at 80 °C.

### GO preparation

GO NPs were synthesized using Hummer’s method. In a beaker, H_2_SO_4_ and H_3_PO_4_ were combined in a 9:1 ratio. One g of graphite powder and 6 g of KMnO_4_ were combined in an ice bath in a different beaker. After that, the two solutions were combined and agitated for 12 h at 50 °C. The mixture was cooled before being transferred into 1 mL of 30% H_2_O_2_ ice. Centrifugation at 10,000 rpm was used to gather the precipitate, which was then cleaned multiple times with deionized water and left to dry overnight in a room temperature vacuum oven. Initially, 200 mL of 30% HCl was used for washing^41^.

### Metal oxide/GO nanocomposites preparation

To prepare metal oxide /GO nanocomposites, the mechanical milling method was employed. 0.06 g of ZnO NPs prepared previously (section “Materials”) and 0.03 g of GO were placed in an agitator mortar. The mixture was then ground for 10 min to create a nanocomposite consisting of ZnO and GO. Similarly, for CuO/GO nanocomposite 0.06 g of CuO NPs prepared previously (section “CuO NPs preparation”) was added to the same percentage of GO, and the grinding process was then carried out for an additional 10 min to yield a CuO/GO nanocomposite.

### CMC/metal oxide/GO nanocomposites preparation

The study employed the solution casting method to synthesis pure CMC and its nanocomposite films. First, 0.5 g of CMC was dissolved in 100 mL of distilled water while stirring continuously for 4 h to create a homogeneous CMC solution. Next, the solution was cooled to room temperature and any air bubbles were removed, and the film solution was cast into two glass petri dishes. The dried films were removed and kept in the sealed bag until the test. This command was used to code the film as CMC.

**Fig. 1:**
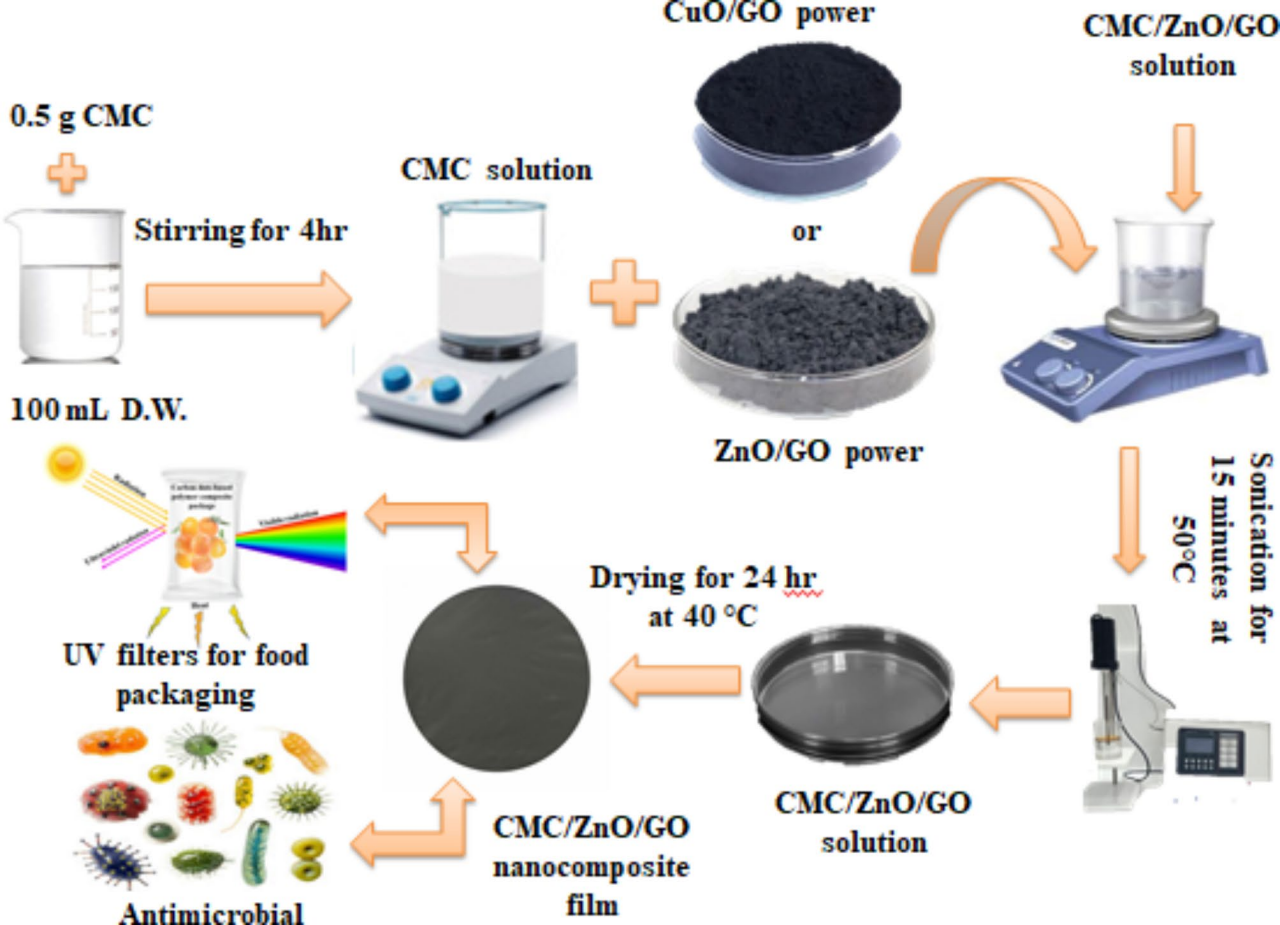
Schematic representation of CMC/ZnO/GO and CMC/CuO/GO nanocomposite synthesis.

The CMC/ZnO/GO and CMC/CuO/GO nanocomposite films were produced by mixing 2 wt% of metal oxide/GO nanocomposite with the previously prepared CMC solution. After 15 min of sonication at 50 °C, the mixture turned dark grey and was transferred into glass petri dishes (see Fig. 1). After pouring the liquid into a glass petri dish and letting it stand for a short while to eliminate any surface bubbles, the liquid was dried at 40 °C in an oven for 24 h to produce the polymer nanocomposite films. The films were then carefully taken out of the petri dishes.

### Characterization techniques

Fourier transform infrared spectroscopy (FTIR, vertex 70, Bruker, Germany) was used to investigate the structure of synthesized nanocomposites and their constituent parts in the range of 4000–400 cm^− 1^. The penetration depth of the diamond ATR accessory with a type II alpha diamond crystal is 2 μm. A total of 35 scans were performed on each spectrum. The same settings were used to test the background versus air with a resolution of 4 cm^− 1^. The CMC nanocomposite films were subjected to X-ray analysis using a Rigaku Smart LabTM (Tokyo, Japan) X-ray diffractometer (XRD) equipped with Cu Kα radiation. The optical properties were investigated in the wavelength range 200–1000 nm using an ultraviolet/visible spectrophotometer (UV/Vis., V-570 UV/VIS/NIR, JASCO, Japan) at room temperature. The nanocomposite films were cut into 2 × 1 cm pieces, and then the pieces were placed inside the UV-Vis Spectrophotometer. The optical background was measured and subtracted from the sample’s spectrum. The thickness of the prepared CMC nanocomposites was measured using a micrometer at five different points on each film, and the average was calculated. The measurements are accurate to ± 0.005 mm. The addition of the ZnO/GO and CuO/GO nanocomposites keeps the CMC thickness (0.46 mm) nearly constant. CMC nanocomposites surface morphology was assessed using scanning electron microscopy (SEM) on a Hitachi S-4800 (Japan) apparatus with an accelerating voltage of 20 kV and equipped with a JEOL-made energy-dispersive X-ray spectrometer (EDS). Prior to testing, the samples were preconditioned at 25 °C. A sonics vibracell (Newtown, CT, USA) ultrasonic processor (500 W, 20 kHz) was used to sonicate the synthesized nanocomposite in CMC at 25% amplitude for 30 min, with 30 s/30 s on/off cycles prior to analysis. The particles were then collected on copper grids coated with carbon. Using a plano-convex lens with a 70 mm focal length and an acoustic optically Q-switched Nd: YAG laser (λ = 1064 nm) with a 7 ns pulse duration, 10 Hz repetition rate, and 50–150 mJ laser fluency per pulse, the plano-convex lens focused the laser beam on the target’s surface.

### Calculation details

It is well known that the most efficient method for examining the electronic characteristics of molecules is DFT. Quantum chemical tools that are widely used to sketch the reactivity and stability of newly synthesized organic compounds are the FMO and the global reactivity descriptor. We primarily used the B3LYP (hybrid) functional for the electronic and optical properties in the DFT analysis.

DFT was utilized to analyze the optimized structures and electronic properties of CMC, GO, CMC/ZnO/GO, and CMC/CuO/GO model molecules. These analyses were conducted using the Gaussian 09 package of programs^42^. The Gauss View 05 program was used to visualize optimized geometries and draw molecular structures^43^. The calculations were performed using Becke’s three-parameter B3 with the Lee, Yang, and Parr (LYP) correlation functional^44–46^. This B3LYP hybrid functional, based on the standard form of the Vosko-Wilk-Nusair correlation potential, incorporates the exchange–correlation functional. Functional B originally contained both the Slater exchange and adjustments pertaining to the density gradient. A correlation functional LYP with both local and nonlocal terms was created by Lee, Yang, and Parr^47^. B3LYP is the most widely used DFT approach because it is capable of accurately predicting molecular structures and other properties. For all structures, the standard LanL2DZ (Los Alamos National Laboratory 2 Double Zeta) basis set was used. The LANL2DZ basis set was used because it is the most effective one in predicting both the ionization potentials and the heats of formation for transition-metal-containing systems, for 94 and 58 systems containing first-row transition metals from Ti to Zn, respectively, all of which are in the third row of the periodic table. It is worth noting that including the exact exchange term in density functional methods improves the accuracy of predicting ionization potential. To comprehend the molecule’s reactivity characteristics, all chemical simulations were run in the gas phase. For analogous organic molecules, the hybrid DFT functional B3LYP in conjunction with the LanL2DZ basis set has been reported earlier^48^.

The stability of the molecular geometry was verified by performing a frequency calculation following optimization. To determine the reactivity and stability of the compound under study, the TDM, frontier molecular orbital (FMO), and the HOMO/LUMO energy gap (the energy difference between the highest occupied molecular orbital (HOMO) and the lowest unoccupied molecular orbital (LUMO)) were computed. In addition, several global reactivity descriptors were computed, such as electron affinity (EA), chemical potential (µ), ionization potential (IP), chemical hardness (η), electronegativity (χ), and electrophilicity index (ω). To acquire additional understanding of the electronic and conductivity properties of the compound under study, the MESP maps were carried out.

### Antibacterial activity of nanocomposite films

#### Agar diffusion test

It was tested against *S. aureus* (ATCC 6538) and *E. coli* (ATCC25922), which represent gram-positive and gram-negative bacteria, respectively. The bacteria were grown in sterile Tryptic Soy and incubated at 37 °C for 24 h to reach the required concentrations. The agar diffusion method, also known as the inhibition zone test, was used to evaluate the films’ antibacterial properties. This method assesses how well the nanocomposite films, which contain metal nanoparticles, release antibacterial agents. If the film releases such agents, a clear zone of inhibited bacterial growth will appear around it. The size of this inhibition zone indicates how much antibacterial material has diffused from the film. Muller Hinton agar culture media were used for both bacteria. After preparing culture plates, 0.1 mL of each bacterial culture was spread onto the medium using a sterilized L-shaped rod, and film samples were placed on the surface. The plates were incubated, and the inhibition zones were measured after 24 h to assess the films’ antibacterial effectiveness^49^.

#### Growth curve of bacteria

Selected bacterial isolates (10% inoculum) and 1% nanocomposite films were cultured in 100 mL muller Hinton broth and incubated in a rotary shaker at 30 °C with a speed of 120 rpm for 72 h. Samples of 5 mL were collected, and the optical density (OD) of the culture was measured at a wavelength of 600 nm using a spectrophotometer^50^.

## Results and discussion

### FTIR analysis

**Fig. 2:**
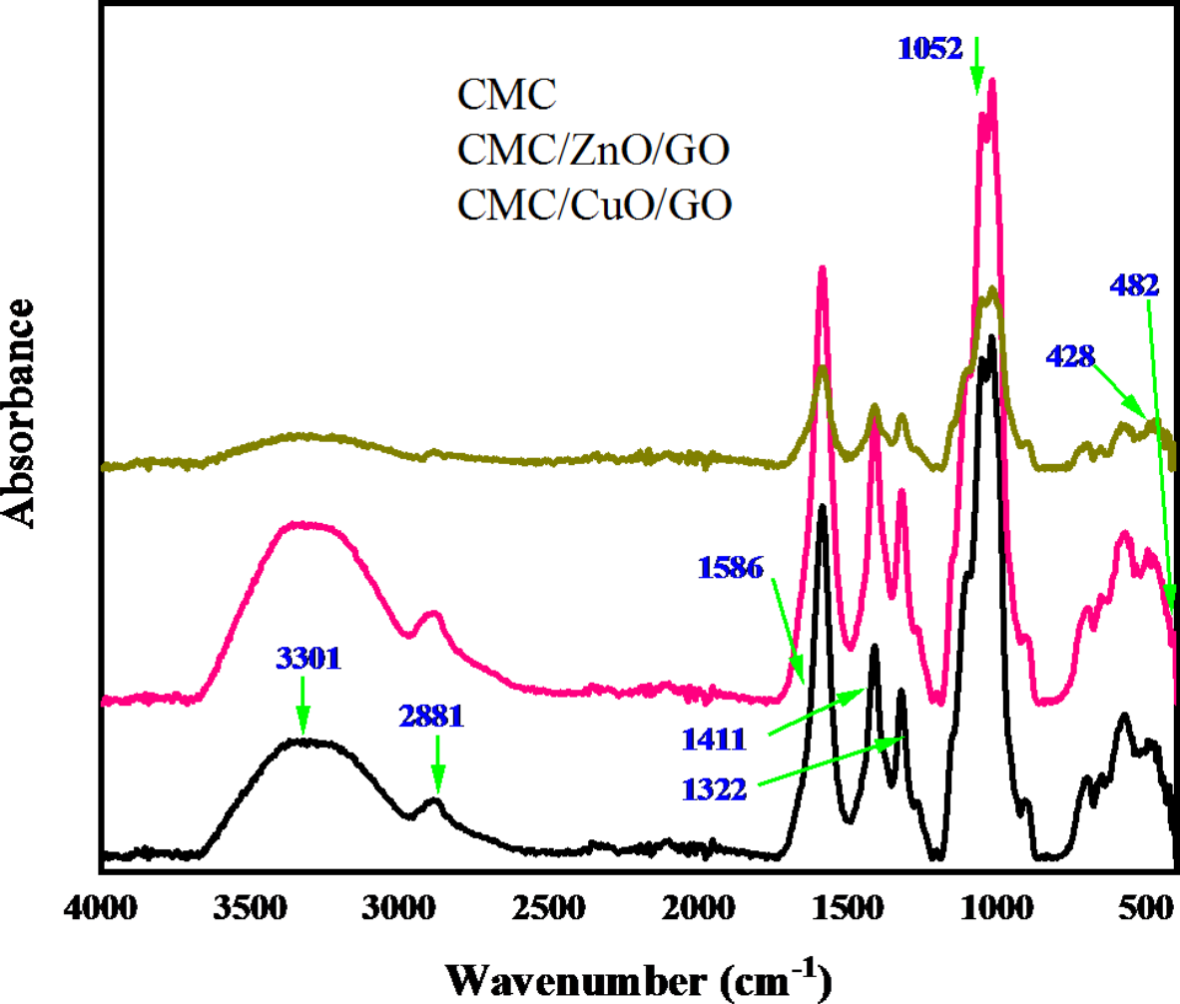
FTIR absorbance spectra of pure CMC and CMC nanocomposite films.

As was previously mentioned, the FTIR spectrophotometer is a very helpful analytical tool for predicting the molecular structure and mode of interaction between the polymeric materials and the nano metal oxides that constitute the samples of nanocomposite materials. The FTIR spectrum of a pristine CMC film, recorded in the 4000:400 cm^− 1^ range, is shown in Fig. 2. Additionally, the spectrum was tabulated and assigned in Table 1. Based on earlier research, the FTIR assignment for the absorption bands was introduced. The hydroxyl group’s (OH) stretching vibration is the cause of the broad absorption band at 3324 cm^− 1^. The band found at 2888 cm^− 1^ is indicative of CH asymmetric stretching. Moreover, the carboxyl group (COO) asymmetric and CH_2_ scissoring are responsible for the sharp absorption bands at 1586 and 1411 cm^− 1^, respectively. Furthermore, the OH and C-O-C bending vibrations are responsible for the 1322 and 1052 cm^− 1^ bands. At last, two absorption bands emerged at 1019 and 912 cm^− 1^, which could be attributed to the CO group of CH_2_OCH_2_ stretching and the C–O stretching + CH_2_ rocking motion, respectively. Apart from the distinct bands of absorption in the 701–572 cm^− 1^ region, these are also ascribed to the stretching and distortion of the α-D-(1–4) and α-D-(1–6) connections^12,13^.

Besides the ZnO and GO characteristic bands, a similar spectrum can be observed in CMC/ZnO/GO and CMC/CuO/GO nanocomposite films. The influence of ZnO/GO and CuO/GO nanocomposite on the CMC molecular structure was also presented in Table 1. The intensity of the CMC characteristic bands increased due to the interaction with ZnO/GO and decreased due to the interaction with CuO/GO nanocomposites as presented in Fig. 2. The CMC’s OH group was shifted to the high wavenumber region at 3325 and 3323 cm^− 1^ due to the interaction with ZnO/GO and CuO/GO nanocomposites, respectively. Meanwhile, the CH stretching vibration was shifted from 2881 cm^− 1^ to 2888 and 2895 cm^− 1^ due to the interaction with ZnO/GO and CuO/GO nanocomposites, respectively. Additionally, the COO asymmetrical stretching band of CMC was shifted to 1590 cm^− 1^ for both nanocomposite films. However, the characteristic bands of O-H and C-H symmetrical stretching observed at 1413 and 1321 cm^− 1^, respectively, are unaffected. C–O stretching + CH_2_ rocking vibration of CMC was shifted to 908 and 900 cm^− 1^ due to the interaction with ZnO/GO and CuO/GO nanocomposites, respectively. The FTIR absorption spectra of CMC/ZnO/GO and CMC/CuO/GO nanocomposite films confirms the complex interaction between CMC and ZnO-NPs as it presents a strong shift of the CMC characteristic bands in addition to a new bands observed in the range of 492 to 414 cm^− 1^ which reflects the presence of ZnO and CuO stretching bands.

**Table 1:** FTIR band assignments for pure CMC and CMC nanocomposites.

Wavenumber (cm^− 1^)			Band assignment
CMC	CMC/ZnO/GO	CMC/CuO/GO	
3301	3325	3323	Stretching of OH group
2881	2888	2895	CH stretching of the CH_2_ groups
1586	1590	1590	Carboxylate group (COO^−^) asymmetric stretching
1411	1410	1412	CH_2_ scissoring
1322	1321	1325	OH bending
1052	1052	1056	The C-O-C bending vibration
1019	1019	1015	C-O bond of the CH_2_OH group
912	908	900	C–O stretching + CH_2_ rocking motion
701 − 572	702 − 572	700 − 572	Ring stretching and deformation
–	482	492	O-Zn-O/ O-Cu-O
–	427	464	O-Zn-O/ O-Cu-O
	414	426	O-Zn-O/ O-Cu-O

### XRD analysis

**Fig. 3:**
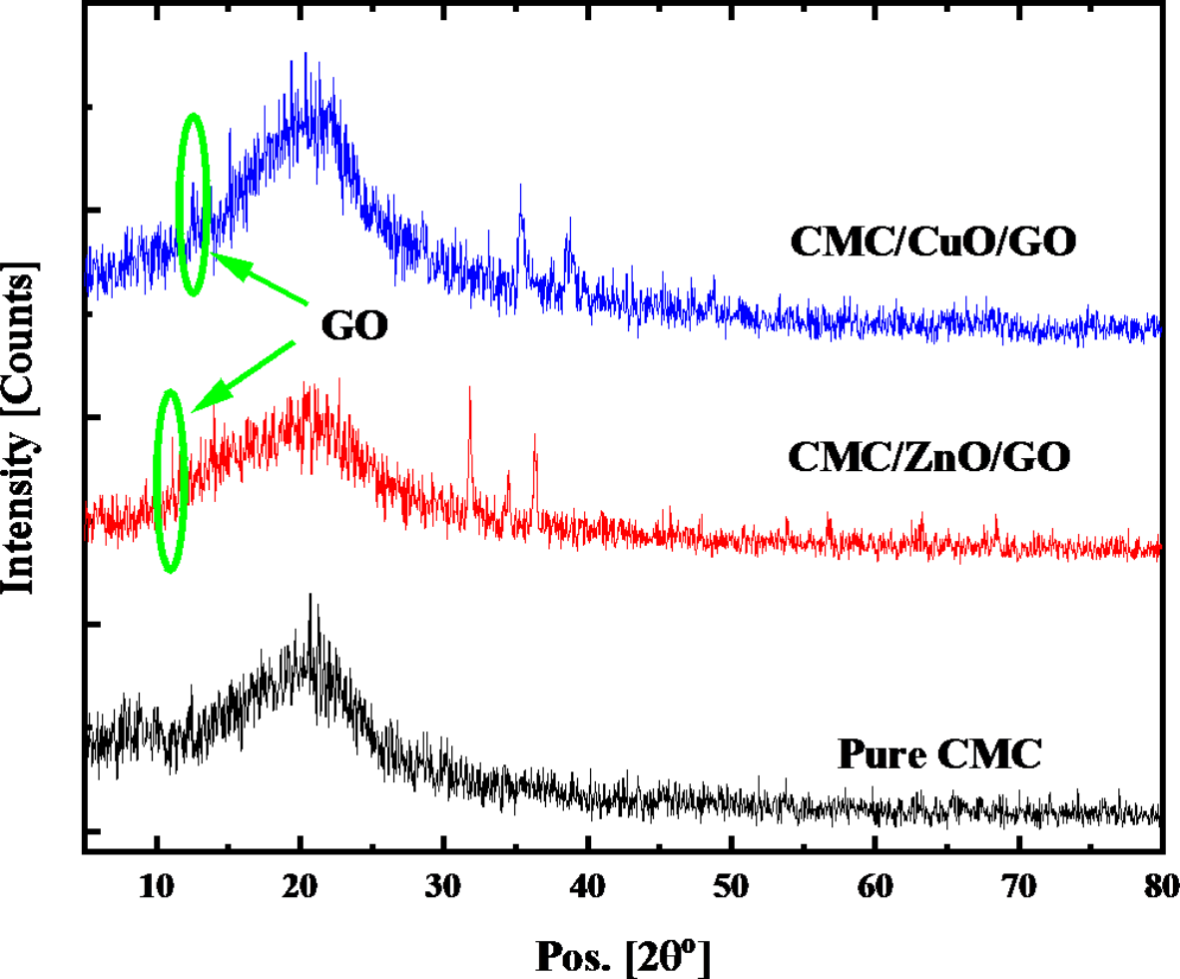
XRD spectra of pure CMC and CMC nanocomposite films.

Only thin-film crystal samples or powder-formed samples can be subjected to the X-ray diffraction (XRD) technique. Crystals can now be classified as either short-range (also known as pseudo or amorphous) or ordered long-range (also known as true) crystals. The sharper and narrower XRD peaks in true crystal samples indicate the various phases that are present in the sample. On the other hand, XRD peaks in amorphous crystals are wider and humpier in shape. The GO XRD peak (low intensity), which corresponds to the (0 0 1) plane, is located between 10° and 11° in this work (see Fig. 3). This result is in good agreement with the previous work, which confirmed that this characteristic peak is located between 7° and 12°^51^.

The X-ray analysis of the virgin CMC film and CMC doped with 2.0 wt% ZnO/GO and CuO/GO is displayed in Fig. 3. The amorphous nature of CMC is reflected by the broad diffraction peak of pure CMC observed at 20°^12,13^. The CMC XRD peak is predominant because of the high miscibility between the amorphous CMC structure and the nanocomposite. Due to the addition of 2.0 wt% of ZnO/GO or CuO/GO nanocomposite, the broadening of the CMC XRD peak increased. This means that the addition of nanofiller causes the CMC’s degree of amorphousness to increase. This result supports the hypothesis that variations in CMC structure result from modifications in the electrostatic interaction between ZnO/GO or CuO/GO nanocomposite and the functional groups of CMC. When 2.0 wt% of ZnO/GO is added, multiple peaks with centers at 31.78°, 36.29°, 56.70°, 3.25°, and 63.25° are produced. These diffraction peaks correspond to the (100), (101), (110), (103), and (201) reflecting planes of ZnO. Additionally, the planes (002), (111), and (–113) for the CuO are represented by the XRD peaks observed at 35.34°, 38.77°, and 61.42°. For ZnO and CuO, these peaks correspond to standard JCPDS cards with numbers 01-078-3315 and 01-078-3315, respectively. Additionally, these peaks reflect the hexagonal and monoclinic structures of ZnO and CuO, respectively, and are in good agreement with our previous work^12,13^.

The CMC nanocomposite films’ crystallite size can be determined using the Scherrer formula.

1 $$~~~~~~{\varvec{D}}=\frac{{{\varvec{K}}\varvec{\lambda}}}{{\varvec{\beta}{\varvec{C}}{\varvec{o}}{\varvec{s}}\varvec{\theta}}}$$

The average size in this case is “D” (Å), and the shape factor for spherical materials is K, 0.93. The angle of XRD is θ, and the full width at half maximum is β. The Cu_kα_ line source’s wavelength, λ, is equal to 1.5406 Å. Half of the 2θ yields the value of θ. By combining these values with other necessary parameters in Eq. (1), the average crystallite sizes of CMC/ZnO/GO and CMC/CuO/GO nanocomposite films were determined to be 42.11 nm and 28.08 nm, respectively.

### Optical analysis

#### Optical absorption

**Fig. 4:**
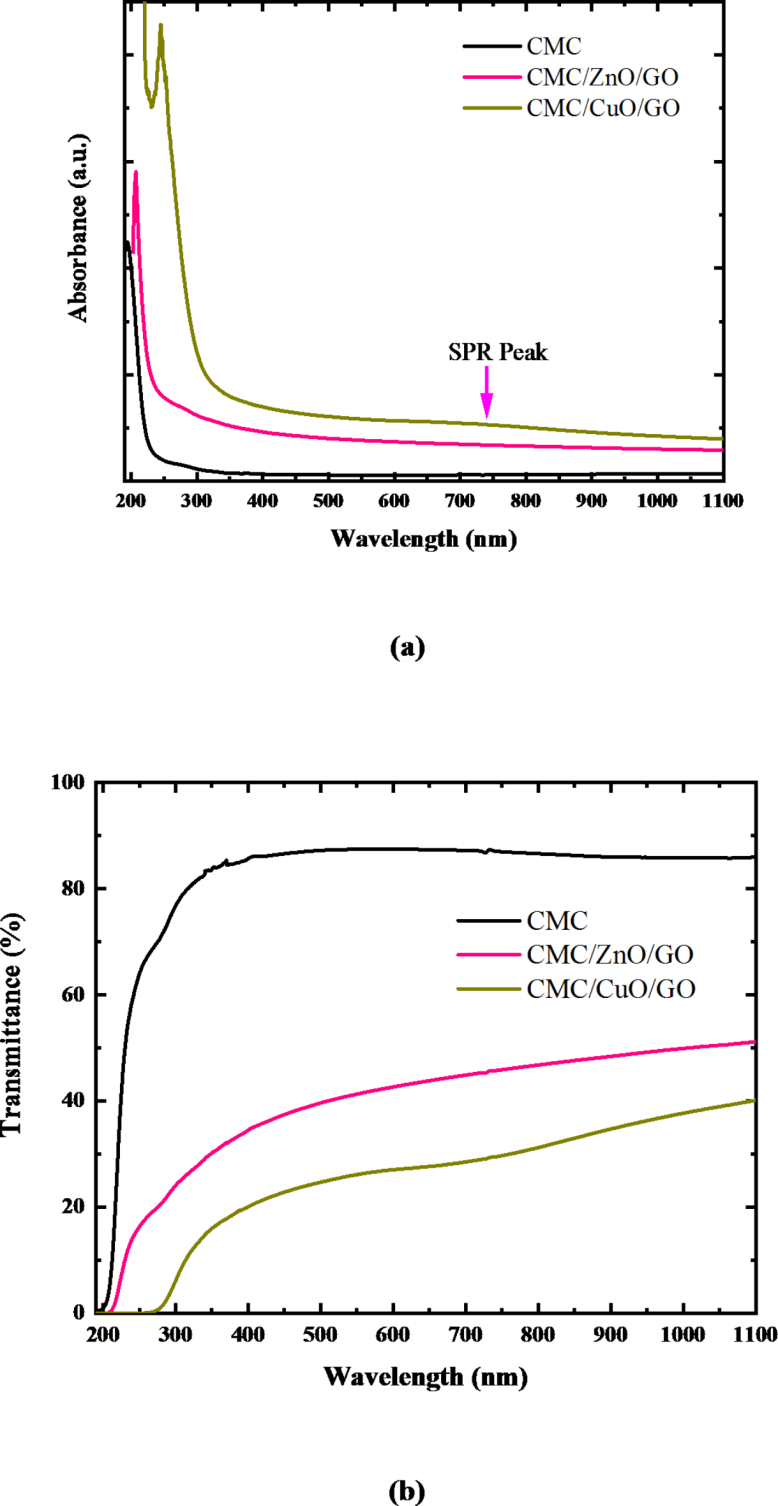
UV-Vis **(a)** absorbance and **(b)** transmittance spectra of pure CMC, CMC/ZnO/GO, and CMC/CuO/GO nanocomposite films.

Determining the optical properties of metal oxides and polymers is very interesting to explore their various applications. The absorbance and transmittance spectra of pure CMC, CMC/ZnO/GO, and CMC/CuO/GO and nanocomposite films are displayed in Fig. 4 along with their wavelengths of collection, which spans the optical range from 200 to 1000 nm.

As can be observed, pure CMC does not exhibit any distinct absorption bands in its UV-Vis spectrum due to its relatively high transmittance in both the UV (200–400 nm) and visible (400–800 nm) regions^12,13^. However, CMC possess an absorption shoulder at nearly 196 nm. This shoulder was shifted to the high wavelength region due to the formation of the nanocomposite films. A broad absorption bands with a wavelength of 207 and 246 nm is visible in the UV–Vis absorption spectrum of CMC/ZnO/GO and CMC/CuO/GO nanocomposite films. The first peak can be attributed to both the CMC and the GO nanoparticles because they overlap in this absorbance range. The characteristic absorption band observed at 207 nm was ascribed to n–π* transitions of C=C functional group and that at 246 nm was attributed to π–π* transition of C=O group which are characteristic for the GO. The CMC/ZnO/GO nanocomposite film has a low intense absorption shoulder at nearly 280 nm belongs to ZnO-NPs. Meanwhile, the broad surface plasmonic resonance (SPR) peak of CuO NPs is observed at 700 nm. The low intensities of ZnO and CuO NP’s characteristic peaks are due to the low concentration of the two nanomaterials in the nanocomposite films. Particle size and the components’ refractive index are examples of intrinsic material properties that affect the absorbance of the nanocomposite. Other factors that affect absorbance during fabrication include the concentration of filler, the thickness of the nanocomposite, the particle dispersion state, and the surface roughness. As a result, particles larger than visible wavelengths would block light, producing films that are either opaque or translucent^51^. The findings of the polymer nanocomposites UV-Vis absorption spectrum verify the existence of ZnO, CuO, and GO NPs in the polymer matrix.

#### UV-shielding performance

The most effective way to comprehend and develop the band gap structure and electronic properties of the polymeric nanocomposite films is to study the UV–Vis–NIR optical absorption and transmittance spectra. Adding ZnO/GO and CuO/GO nanocomposites results in a significant decrease in the optical transmittance, as shown in Fig. 4-b. This is due to the higher optical absorption edge. This indicates a significant interaction between CMC and ZnO/GO and CuO/GO inorganic nanocomposites. The transmittance spectrum is divided into two regions: transparent with low absorption in the visible region and a zone of strong absorption in the UV region, where transmittance decreases significantly due to the absorption coefficient.

As presented in our previous work, the addition of CuO-NPs to the CMC matrix had a significant impact on its ability to absorb UV-light^12^. Moreover, CuO NPs addition to the CMC solution improved CMC’s UV-blocking performance, particularly in the UV-A area^13^. Table 2 compares published studies on the UV blocking capabilities of various transparent polymer films, including the current study. The table shows how 8 wt% CuO NPs additions can block 99% of UV-B (280–320 nm) and UV-A (320–400 nm) radiation.

In the UV-Vis region, pure CMC has an optical transmittance of approximately 88% and blocks approximately 15% in the UV-A region. On shorter wavelengths, transmittance with incident light decreased, which can be attributed to the layer formed by intermolecular hydrogen interaction between the pure CMC matrix and ZnO/GO and CuO/GO nanocomposites. The CMC/ZnO/GO film has a transmittance in the UV-C region of 20%, transmittance in the UV-B region equal 26%, and transmittance in the UV-A is 34%.

According to our previous work^12^, the addition of 2 wt% CuO NPs only blocks 66% of UV-B (280–320 nm) and 52% of UV-A (320–400 nm) radiation. However, the prepared CMC/CuO/GO nanocomposites film completely blocks light in the UV-C region (200–280 nm), even though the UV-B and UV-A regions were not completely blocked. CMC/CuO/GO nanocomposite film transmits only 11 and 20% in the UV-B and UV-A regions, respectively. This means that the addition of 1 wt% of GO contributes strongly to the improved UV absorption and results in the observed increase in the UV blocking percentage, the decrease in the optical bandgap energy, and hence the increase in the Urbach energy. This is due to the strong absorption of GO in the UV spectrum since GO peak has been observed previously at 230 nm and shoulder at 300 nm. The ability of CMC/CuO/GO nanocomposite films to function as broadband UV-Vis filters is confirmed by raising the blocking percentage to 89% and 80% in the UV-B and UV-A regions, respectively. Our results thus qualify CuO/GO filled CMC nanocomposite films as promising choices for various optical and storage applications, including UV radiation blocking. These findings were in line with a prior study that found that adding lignin to cellulose-based films increases UV protection while decreasing transparency^50^. This can also demonstrate that each material was a potential candidate for UV-shielding applications, which are preferred in the food packaging industry.

**Table 2:** UV blocking percentage of CMC and CMC nanocomposite films.

Sample	UV Blocking %			Opacity	T_600_%	Ref.
	UV-C	UV-B	UV-A			
CMC	29	20	15	1.26	4.22	Current study
CMC/2wt.% ZnO/GO	80	74	66	7.79	3.43	Current study
CMC/2wt.% CuO/GO	100	89	80	12.21	3.08	Current study
CMC/8%CuO	100	99	99			^12^
CMC/4% CuO@ZnO core/shell	100	100	100			^13^
CMC/lignin	–	98	89			^52^
PMMA/ 0.05% ZnO quantum dots	100	100	50			^53^
PVA/ 5% Dopamine−Melanin	100	100	70			^54^

Table 2 shows the relationship between opacity and the nanofiller for pure CMC and CMC nanocomposite films at 600 nm. The film opacity was calculated using Eq. (2)^13^:

2 $${\varvec{O}}{\varvec{p}}{\varvec{a}}{\varvec{c}}{\varvec{i}}{\varvec{t}}{\varvec{y}}=~\frac{{{{\varvec{A}}_{600}}}}{{\varvec{d}}}$$

Where, d is the film thickness (mm) and A_600_ is the absorbance at 600 nm. A film is less transparent if its opacity number is higher. The prepared films can be easily observed to exhibit a gradual change in colour from transparent to relatively dark grey, with the CMC film having a lower opacity value (1.26) and being more transparent than the nanocomposite films.

By measuring the percent light transmittance (%T) at 600 nm, the transparency was obtained. The transparency (T_600_) was computed using the following equation:

3 $${{\mathbf{T}}_{{\mathbf{600}}}}=\frac{{{\mathbf{Log}}~\left( {{\mathbf{T}}\% } \right)~{\mathbf{x}}~100}}{{\mathbf{d}}}$$

Where, d is the film thickness (mm). As presented in Table 2, the transparency of CMC decreased from 4.222 to 3.431 and 3.080 due to the interaction with ZnO/GO and CuO/GO nanocomposite, respectively. The transparency of CMC film containing ZnO NPs was higher than that of the film containing CuO NPs. The observed decrease in film transparency is the consequence of light transmission being obstructed by the opaque appearance of CMC nanocomposite films.

#### Absorption parameters and refractive index determination

The Beer-Lambert formula can be used to calculate the absorption coefficient α.

4 $$\varvec{\alpha} ={\mathbf{2}}.{\mathbf{303}}\frac{{\varvec{A}}}{{\varvec{d}}}$$

**Fig. 5:**
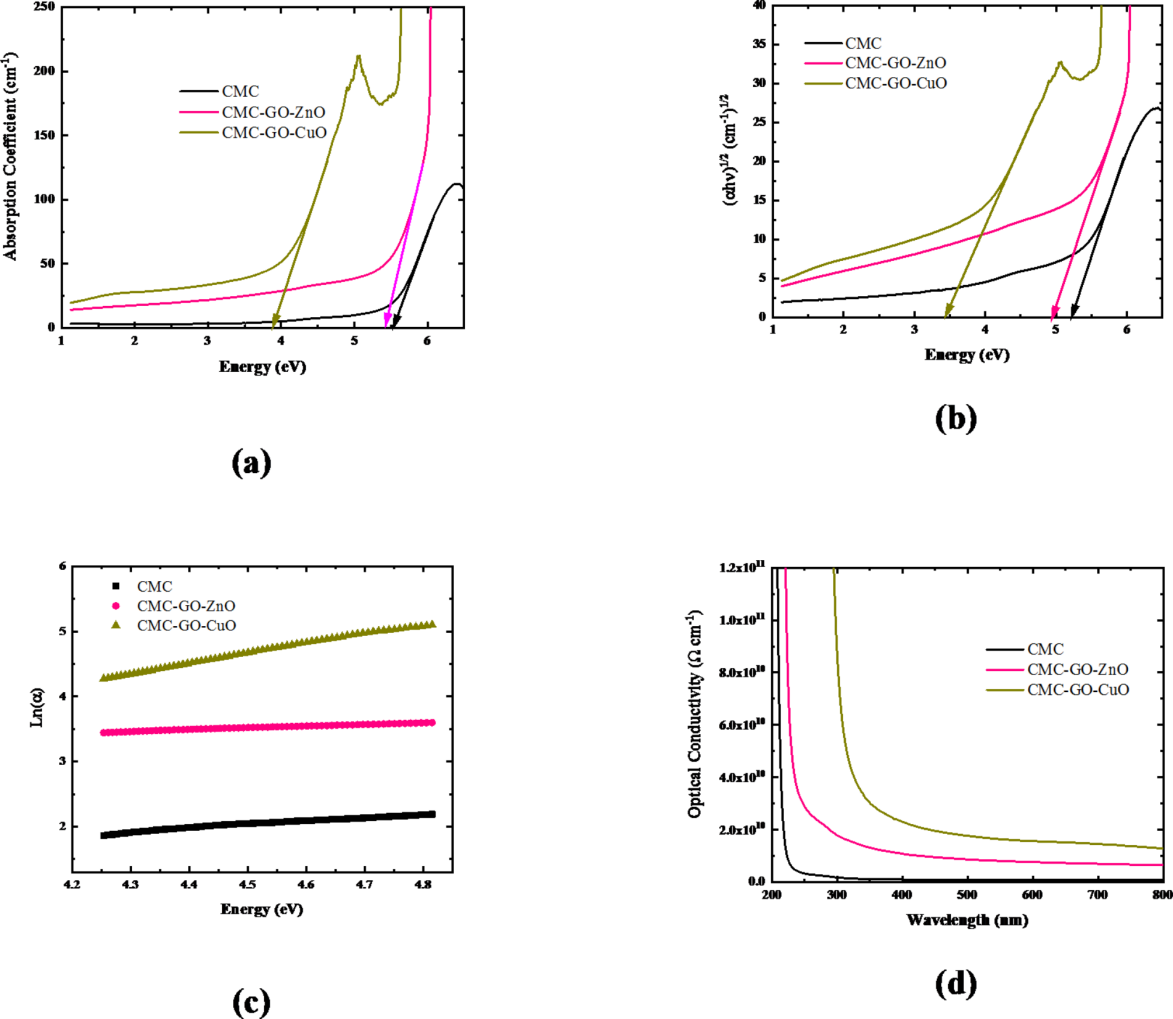
Variation of **(a)** absorption coefficient, **(b)** (αhv)^1/2^, **(c)** Lin α with incident photon energy, and **(d)** optical conductivity with wavelength for CMC, CMC/ZnO/GO, and CMC/CuO/GO nanocomposite films.

Where A is the absorbance of the film. The change in the absorption coefficient with incident light for both pure and nanocomposite CMC films is shown in Fig. 5-a. It is evident that the pure CMC film exhibits a high degree of amorphousness by its significant absorption in the UV spectral region. The location of the absorption edge was found by plotting the coefficient of absorption for each nanocomposite film against the photon energy (hv), as shown in Fig. 5-a. The value of the fundamental edge can be obtained by extrapolating the linear portion of the curve to the energy axis for each sample. By varying the metal oxide in the CMC nanocomposite matrix, one can observe a continuous red shift in the graph. Table 3 presents the variation of the absorption edge of CMC due to the interaction with ZnO/GO and CuO/GO nanocomposites.

According to Mott and Davis^12^, the following formula can be used to determine the optical gap E_g_ for non-crystalline materials:

5 $$({\mathbf{ahv}})\,=\,{\mathbf{B}}{({\mathbf{h}}\nu - {\mathbf{Eg}})^{\mathbf{r}}}$$

Where, r is a constant that depends on the type of transition; *r* = 1/2 for allowed direct transitions and 3/2 for forbidden direct transitions, and *r* = 2 and 3 for indirect allowed and forbidden transitions, respectively. In addition, h is the plank’s constant, ν is the incident frequency, and B is a constant.

Based on our previous work, it’s found that CMC has an indirect band gap^12^. Accordingly, the relationship between (αhυ)^1/2^ versus photon energy is depicted in Fig. 5-c for pure CMC and CMC nanocomposite films. The optical energy gaps were determined by extending the linear portion of the high energy curve of the y-axis to intersect the x-axis. According to Fig. 5-b, pure CMC had an indirect optical gap ($\:{E}_{g}^{in}$) of 5.23 eV, which is in good agreement with the reported data^12^. During the process of filling the CMC with ZnO/GO and CuO/GO nanocomposites, the HOMO/LUMO bandgap of the CMC decreases from 5.23 eV to 4.94 and 3.43 eV, respectively. The improvements in optical properties were attributed to changes in the polymeric structure caused by the addition of ZnO/GO and CuO/GO nanocomposites, which increased the number of defects. Furthermore, the improvement in optical properties could be explained by increasing the degree of disorder caused by the produced localized states inside the CMC energy gap, as the introduction of ZnO/GO and CuO/GO nanocomposites in the polymer may generate energy levels inside the CMCs’ energy gap, causing the optical energy gap to narrow^55^. These tunable optical properties of CMC nanocomposites make them promise for various applications.

The values of $E_{g}^{{in}}$ are tabulated in Table 3. Moreover, this decrease can be explained by the formation of hydrogen bonds between ZnO/GO and CuO/GO nanocomposites and the pure CMC, as was previously mentioned in the FTIR section. Indirect band gap semiconductor materials are excellent choices for UV-blocking applications due to the momentum conservation law. The moments of electrons and holes differ in indirect semiconductors. They must therefore do something with this uncompensated momentum in order to recombinantly fulfil the momentum conservation law. In direct band gap semiconductors, electron and hole-pair momentum can be zero, enabling simple recombination.

Additionally, according to our previous work^12,13^, increasing the NPs concentration could improve the optical properties of polymeric materials. As the increased concentration of CuO and CuO@ZnO core/shell NPs results in a decrease in the optical bandgap energy (both onset and HOMO/LUMO gaps), an increase in Urbach energy, a decrease in transmittance percent, a decrease in oscillation energy, an increase in dispersion energy, infinite and lattice dielectric constants, and free carrier concentration. Additionally, the nanocomposite films loaded with different concentrations of NPs will possess a high blocking capacity of the UV radiation, making them suitable candidates for UV blocking applications. Optical experiments, reported previously^12,13^, revealed that pure CMC films exhibited varying transmittance levels within the UVC, UVB, and UVA ranges. Increasing the concentration of NPs led to a reduction in the CMC’s transmittance within the UVC, UVB, and UVA ranges.

The reduction of the absorption edge supports the emergence of localized states in the prohibited band and the rise of non-crystalline ordering in the CMC film by ZnO/GO and CuO/GO nanocomposites. This can be demonstrated by calculating the Urbach energy (E_u_), which indicates the width of the localized states inside the forbidden gap, using the following formula^13^:

6 $${\mathbf{\alpha }}\,=\,{{\mathbf{\alpha }}_{\mathbf{o}}}{{\varvec{e}}^{\left( {\frac{{{{\varvec{E}}_{\varvec{U}}}}}{{{\varvec{h}}\varvec{\upsilon}}}} \right)}}$$

Where α_o_ is a constant. Figure 5-c displays the Urbach plot of the pure and doped CMC samples. The tailing of the density of states in the forbidden energy gap has been attributed to the lack of long-range order in amorphous materials^12,13^. The photon absorption is linked to the existence of localized tail states in the forbidden gap. Equation (6) is used to calculate the defect levels in the gap, which are reflected in the width of the Urbach tail.

The optical bandgap is inversely proportional to the Urbach energy. Table 3 illustrates how the values of E_u_ increased due to the nanocomposite addition. Reportedly, increased E_u_ values are linked to the increment of the structural instability and disorders in polymer nanocomposites. There will be more opportunities for transitions from band to tail and tail to tail as a result of the states being moved from band to tail. However, a decrease in the optical bandgap is probable.

The refractive index (n) values for pure CMC and CMC nanocomposite samples were calculated using the following equation^56^:

7 $$\frac{{{{\varvec{n}}^2} - 1}}{{{{\varvec{n}}^2}+1}}=1 - \sqrt {\frac{{{{\varvec{E}}_{\varvec{g}}}}}{{20}}}$$

Table 3 displays the observed n values for these polymer nanocomposite materials, which are 1.71, 1.73, and 1.97 for pure CMC, CMC/ZnO/GO, and CMC/CuO/GO nanocomposite films, respectively. Additionally, the n values show a nonlinear increase with ZnO/GO and CuO/GO nanocomposites, indicating a modification in the packing densities and an enhancement in the interatomic spacing of these materials.

The optical conductivity is calculated by using the following equation^55^:

8 $$\sigma = \frac{{~\alpha nc}}{{4\pi }}$$

Figure 5-d depicts the variation of CMC nanocomposites’ optical conductivity with wavelength. The electrical conductivity resulting from the charge carriers’ movement caused by the incident electromagnetic waves alternating electric field is referred to as “optical conductivity.” It has been observed that as the CMC interacted with metal oxide/GO nanocomposites, so did the optical conductivity increase. Additionally, the results show that the nanocomposite film based on CuO NPs possesses the highest conductivity. Table 3 presents the optical conductivity at 600 nm for CMC and CMC nanocomposites. The results showed that the conductivity of CMC increased from 8.26 × 10^8^ Ω cm^− 1^ to 1.56 × 10^10^ Ω cm^− 1^ for CMC/CuO/GO nanocomposite film. The decrease in the optical band gap caused by the formation of new levels makes it easier for electrons to cross from the valence band to these local levels and enter the conduction band, resulting in a reduction in the band gap and an increase in the optical conductivity. Additionally, as presented in Table 3, the thickness of the CMC nanocomposite films is nearly constant. This means that the significant changes in the CMC’s optical properties do not depend on the thickness of the CMC’s films but on the filler type. From Table 3, it’s clear that the optical band gap decreased with decreasing the crystallite size. This is due to the dislocations of atoms from their lattice sites, hence increasing the lattice defects and facilitating the low energy transitions^12^.

**Table 3:** Absorption edge, indirect band gap, Urbach energy, refractive index and optical conductivity of CMC and CMC nanocomposites.

Sample	Thickness (mm)	Absorption edge (eV)	${\text{E}}_{{\text{g}}}^{{{\text{in}}}}$ (eV)	E_u_ (eV)	n	σ (Ω cm^− 1^)	Crystallite size (nm)
CMC	0.46	5.53	5.23	0.37	1.71	8.26 × 10^8^	
CMC/ZnO/GO	0.47	5.41	4.94	0.53	1.73	7.64 × 10^9^	42.11
CMC/CuO/GO	0.46	3.88	3.43	0.66	1.97	1.56 × 10^10^	28.08

### SEM analysis

The SEM images can demonstrate how the ZnO and CuO NPs are distributed and arranged, as well as whether the GO contains any fillers or additives. SEM images can shed light on the dispersion of ZnO/GO and CuO/GO nanocomposites within the CMC matrix as well as the interaction between the nanocomposites and the polymer matrix. The images can also demonstrate how the nanocomposites affect the CMC’s general morphology and structure. Researchers can better understand the microstructure and characteristics of CMC matrix and their nanocomposites by examining the SEM images.

**Fig. 6:**
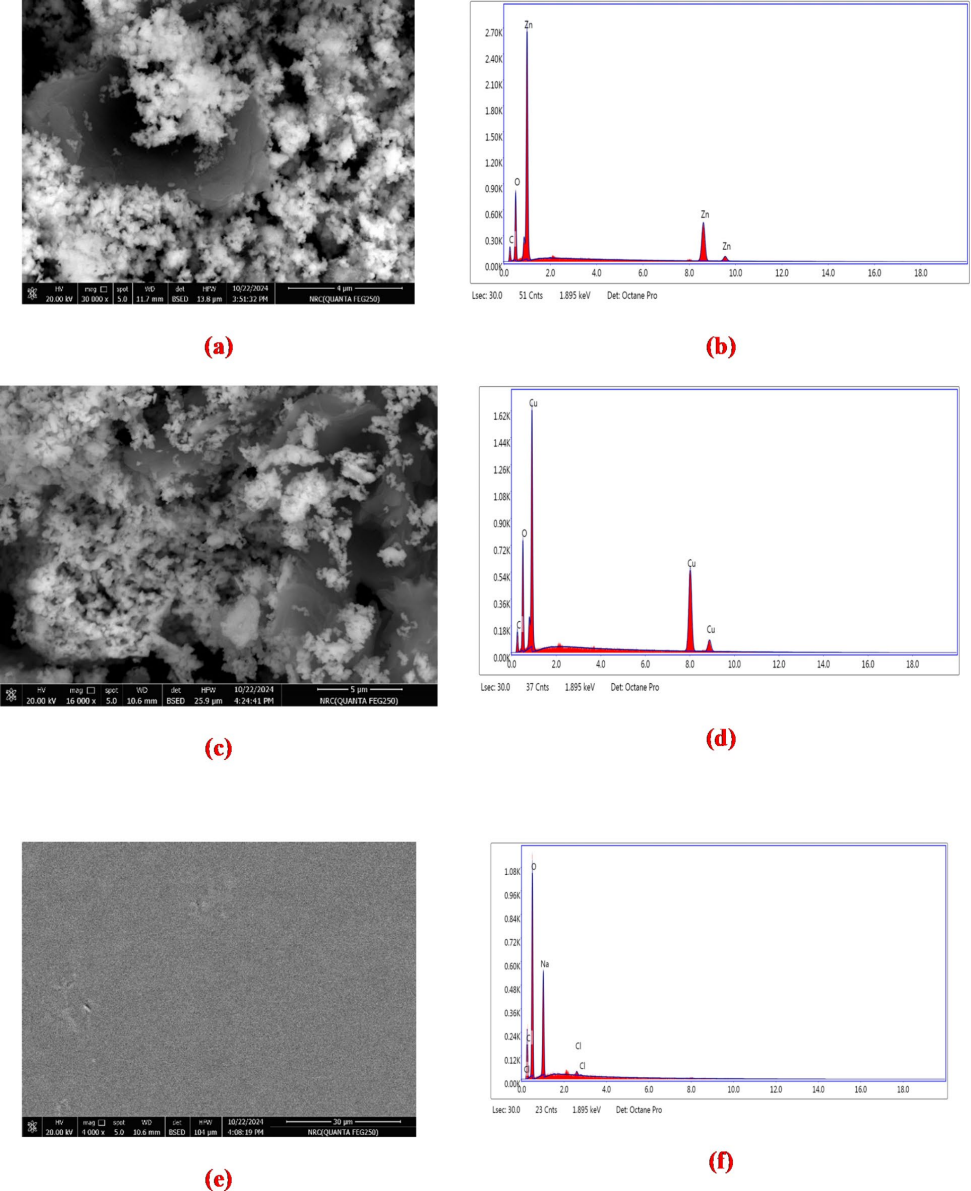

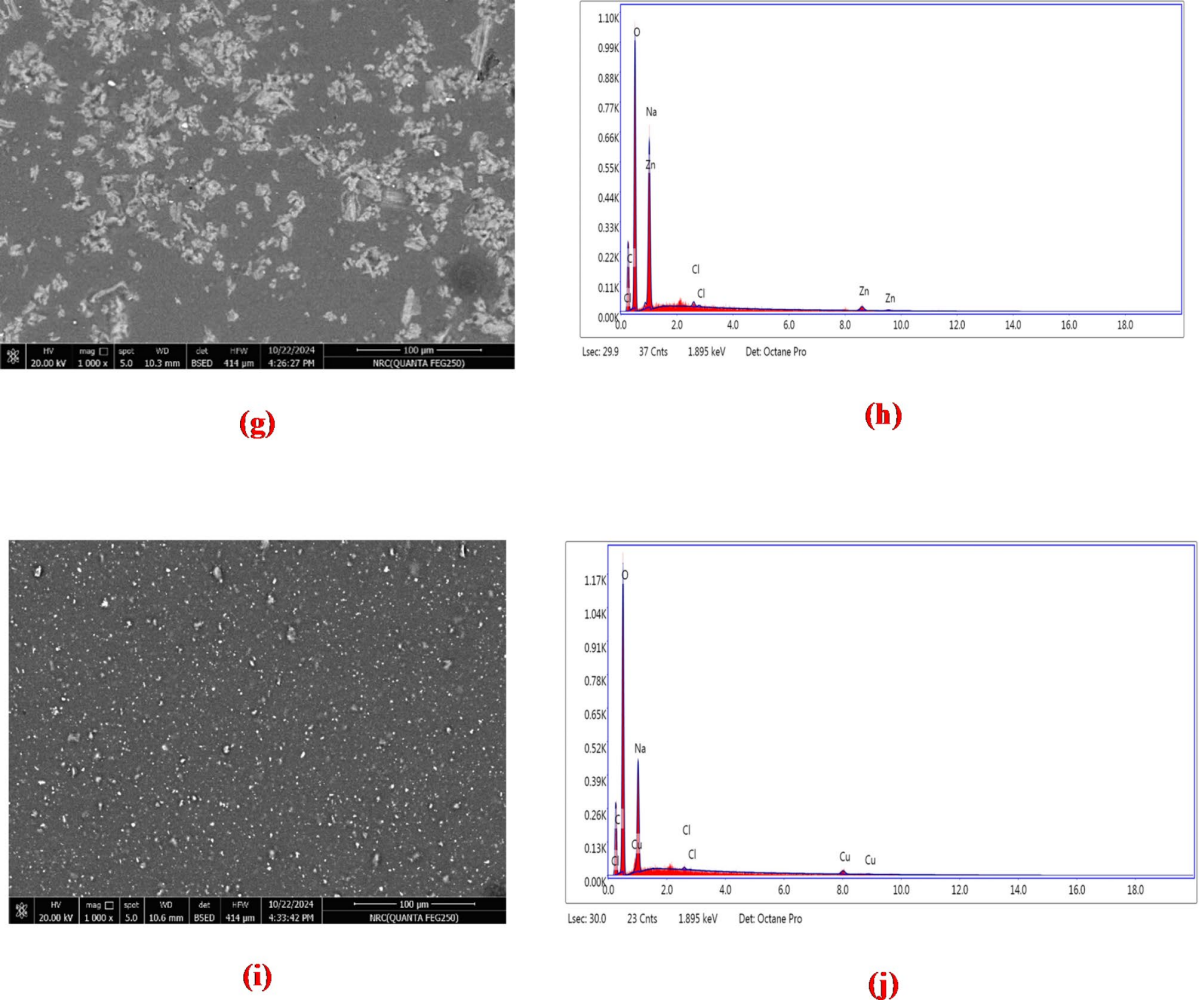
Scanning electron microscope (SEM) image of (a) ZnO/GO (scale by 4 μm), (c) CuO/GO (scale by 5 μm), (e) pure CMC (scale by 30 μm), (g) CMC/ZnO/GO (scale by 100 μm), and (i) CMC/CuO/GO nanocomposites (scale bar 100 μm) and the elemental mapping analysis of (b) ZnO/GO, (d) CuO/GO, (f) pure CMC, (h) CMC/ZnO/GO, and (j) CMC/CuO/GO nanocomposites.

Figure 6 depicts SEM images taken to investigate the surface morphology of ZnO/GO, CuO/GO, CMC/ZnO/GO, and CMC/CuO/GO nanocomposites. Figure 6a and c show ZnO and CuO NPs that have been dispersed on GO sheets, respectively. It is observed that both ZnO and CuO NPs have agglomerated. Figure 6-e shows the SEM image of pure CMC. The results demonstrated the semi-crystalline nature of CMC with flattened texture in the cross-sectional image.

The developed CMC film’s morphology was seen to be a rough surface. The SEM image of the CMC/ZnO/GO film (Fig. 6-g) revealed enhanced roughness with tiny GO sheets and observable spherical ZnO NPs. Nonetheless, the CMC/ZnO/GO composite film exhibits some aggregation and larger ZnO NPs sizes. Furthermore, the findings demonstrated that the CuO NPs were evenly distributed throughout the CMC matrix (Fig. 6i). Higher stability in dispersions and lower phase separation lead to higher quality of the CMC/CuO/GO nanocomposite films, showing greater absorbance and homogeneity in the UV-Vis-near IR spectrum. The interaction of ZnO/GO and CuO/GO NPs with various groups of the CMC components was demonstrated by the observed roughness of the developed films. Finally, the SEM images of ZnO/GO and CuO/GO show ZnO and CuO NPs closely adhered to the surface of GO. The EDX analysis reveals the presence of Zn and Cu in a large fraction, which corresponds to the nanoparticles added for the decoration of the GO, while the primary constituent elements are carbon and oxygen. This ratio increased due to the formation of the polymer nanocomposite.

**Table 4:** The Energy-dispersive X-ray (EDX) element composition analysis for ZnO/GO, CuO/GO, pure CMC, CMC/ZnO/GO, and CMC/CuO/GO nanocomposites.

Sample	Element	K ratio	wt%
ZnO/GO	C	0.0370	17.33
	O	0.0834	27.66
	Zn	0.4735	55.01
Total			100
CuO/GO	C	0.0396	11.20
	Cu	0.4857	47.10
	O	0.0000	41.70
Total			100
CMC	C	0.0977	20.73
	Na	0.0735	17.49
	Cl	0.0040	0.38
	O	0.0000	61.40
Total			100
CMC/ZnO/GO	C	0.0987	20.84
	Na	0.0650	15.72
	Cl	0.0034	0.32
	Zn	0.0205	1.65
	O	0.0000	61.47
Total			100
CMC/CuO/GO	C	0.1028	21.45
	Na	0.0555	14.38
	Cl	0.0016	0.16
	Cu	0.0172	1.46
	O	0.0000	62.56
Total			100

### Electronic properties

#### Frontier molecular orbital analysis

**Fig. 7:**
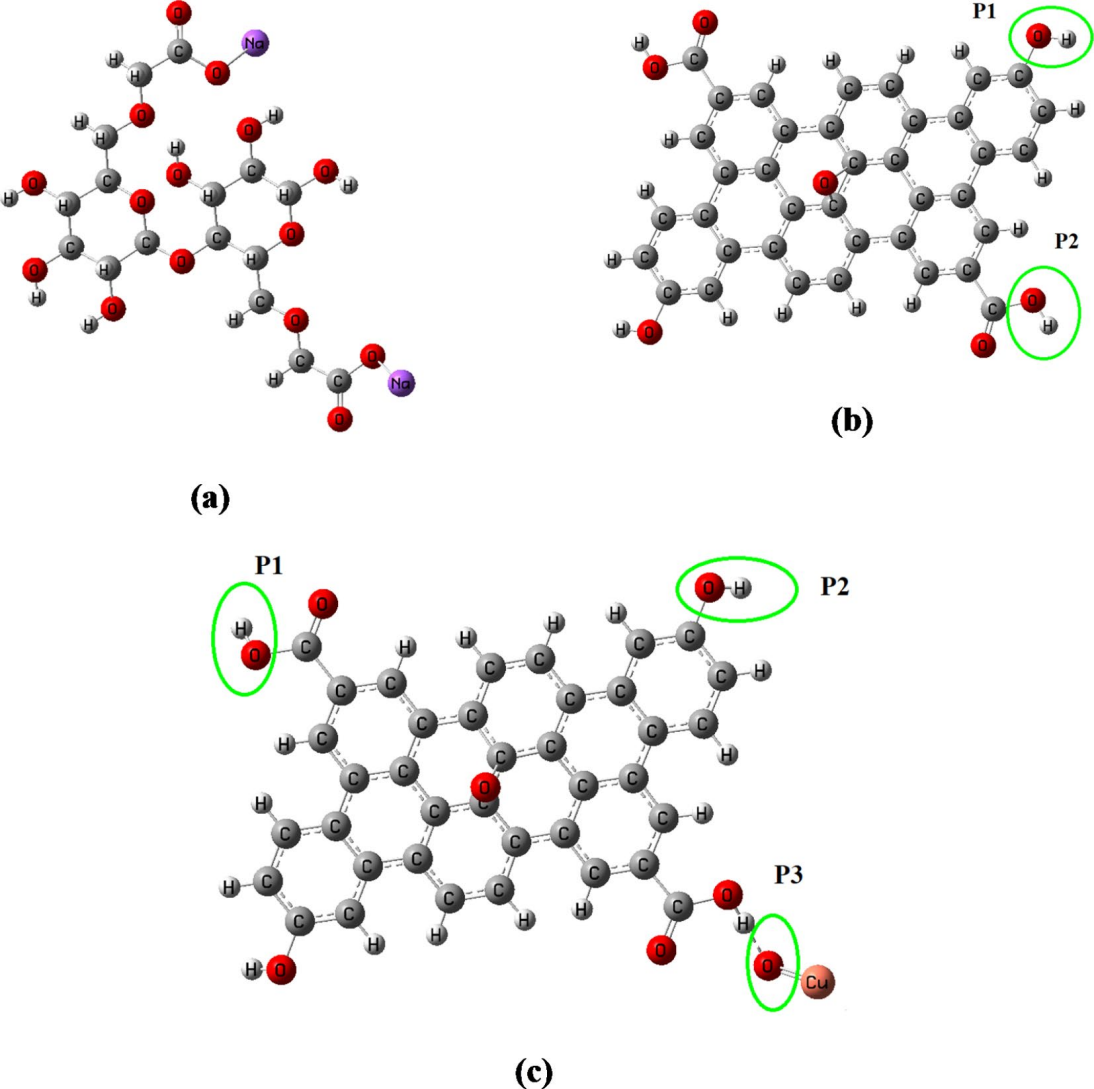
Optimized structures of (a) CMC, (b) GO with two active sides for interaction with metal oxides, (c) CuO/GO with the three active sides for interaction with CMC.

To support the experimental results from FTIR and optical bandgap analyses and to describe the framework of connection between the CMC and the GO nanocomposites, model molecules representing CMC and its interaction with ZnO/GO and CuO/GO nanocomposites were examined. A model of one unit of CMC interacting with a GO sheet with 38 carbon atoms decorated with one molecule of ZnO or CuO was created. According to earlier research, the interaction between GO and the studied CMC or metal oxides mostly happens between the hydrogen atoms of the hydroxyl and carboxyl groups on GO and OH groups of CMC and the oxygen atom of the metal oxide^17,19^. As a result, as shown in Fig. 7, we investigated two possible interaction sites for GO interacted with ZnO and CuO.

Based on the TDM and HOMO/LUMO band gap energy results, the GO/metal oxide structure that possesses the lowest energy was chosen to interact with CMC.

The CMC chain’s terminal interacts with the one possible site of GO/metal oxide model molecule. DFT was used to optimize these models at the B3LYP/LanL2DZ level. The frequency calculations analysis show that the structure is a true minimum on the potential energy surface with no imaginary frequency associated.

A useful theoretical method for examining the electronic and optical characteristics, kinetic stability, and reactivity of synthesized nanocomposites is TDM, frontier molecular orbital (FMO) analysis. The energy of the highest occupied unoccupied molecular orbitals (E_HOMO_), the energy of the lowest unoccupied molecular orbitals (E_LUMO_), and their HOMO-LUMO energy gaps (ΔE) are all included in the FMO study. Small HOMO-LUMO gaps are typically associated with softness and increased reactivity in molecules. Better optical qualities and less kinetic stability are also indicated by this.

The results show that the CMC model has a TDM of 6.151 Debye, HOMO energy of − 2.013 eV, LUMO energy of − 1.415 eV, and $\:\varDelta\:$E of 0.598 eV. Meanwhile, the supposed structure of GO has a TDM of 1.456 Debye, HOMO energy of − 5.099 eV, LUMO energy of − 3.536 eV, and $\:\varDelta\:$E of 1.563 eV. Additionally, TDM increased to 6.411 and 6.836 Debye due to the interaction of GO with ZnO and to 5.341 and 8.170 Debye due to that occurred with CuO through the two interaction mechanisms, respectively.

Because the model representing CuO/GO interacted throughout the COOH functional group of GO has the lowest bandgap energy, we can start from this level to study its interaction with the supposed CMC structure.

There are three probabilities for the interaction of CMC with CuO/GO model molecule. The first probability is that CMC interacts with CuO/GO model throughout the hydrogen atom of the COOH group. Meanwhile, the second and third probabilities of the interaction are those proceeds through the OH group and oxygen atom of CuO, respectively. As presented in Table 5, the CMC reactivity enhanced strongly due to its interaction with CuO/GO. Additionally, the results show that reactivity depends strongly on the interaction site. The TDM of CMC increased to 84.031, 51.432, and 30.071 Debye for the three probabilities of the interaction, respectively. The difference in TDM with the interaction site confirms the hydrogen bond formation between CMC and CuO/GO nanocomposites and highlights the IR activity of these models. These results clearly show that the addition of CuO/GO improves the structural, optical, and electronic properties of CMC and are in good agreement with the FTIR results.

The asymmetric distribution of charges inside the molecule is indicated by the nonzero dipole moment. The total of the individual bond dipoles is the measured TDM. The increased TDM of GO due to the interaction with ZnO and CuO confirms the increased Polarizability.

Additionally, the results show that model’s HOMO and LUMO energies are influenced due to the interaction with ZnO and CuO. The higher HOMO and LUMO energies have reduced the HOMO-LUMO gap by approximately 11%. As a result, the studied compound has small energy gaps and qualifies as reactive organic materials. As presented in Table 5, the structure representing GO interacted with CuO has the lowest bandgap energy in comparison with that of ZnO. Additionally, the interaction proceeds through the COOH functional group is the most probable due to its minimum energy required to excite an electron from the HOMO to the LUMO.

For CMC interacted with CuO/GO, the HOMO/LUMO band gap changed to 0.118, 0.321, and 1.014 eV for the interaction proceeds through the COOH group, OH group, and O of CuO, respectively.

It’s well known that the molecules undergo a change in their TDM are IR active; in this work, the TDM of the supposed structures changes with changing the interaction site. Additionally, the interaction proceeds through the hydrogen atom of the COOH functional group possesses the highest TDM value, which means that CMC can interact with the CuO/GO model more probably through this functional group. This result confirmed the FTIR result in which the strong shift occurred in the COOH asymmetric stretching band of CMC due to the addition of CuO/GO nanocomposite.

Furthermore, the results show that the interaction proceeds through the COOH functional group, which is the most probable as it has the lowest band gap energy of 0.118 eV. This result confirmed the experimental results of UV-Vis analysis that the optical band gap of CMC decreased due to the formation of polymer nanocomposites and that the CMC/CuO/GO nanocomposite film has the lowest optical band gap. Therefore, it can be concluded that the DFT provides a more convenient way to predict interaction mechanisms and confirm the optical results.

**Table 5:** B3LYP/Lanl2DZ calculated total dipole moment (TDM) as Debye, HOMO energy, LUMO energy, and HOMO/LUMO bandgap energy ($\:\varDelta\:$E) as eV for CMC, GO, ZnO/GO, CuO/GO, and CMC/CuO/GO model molecules.

Structure	TDM (Debye)	E_HOMO_ (eV)	E_LUMO_ (eV)	$\:\varDelta\:$E (eV)
CMC	6.151	− 2.013	− 1.415	0.598
GO	1.456	− 5.099	− 3.536	1.563
GO (OH)- ZnO	6.411	− 5.056	− 4.668	0.389
GO -(COOH)- ZnO	6.836	− 5.032	− 4.471	0.561
GO -(OH)- CuO	5.341	− 5.078	− 4.584	0.494
GO -(COOH)- CuO	8.170	− 4.985	− 4.611	0.374
CMC-(COOH)CuO-GO (P1)	84.031	− 2.505	− 2.387	0.118
CMC-(OH) CuO - GO (P2)	51.432	− 3.338	− 3.017	0.321
CMC- (OCu) CuO- GO (P3)	30.071	− 4.017	− 3.002	1.014

#### Global reactivity descriptors

Fundamentally, chemical reactivity is the study of a molecule’s reaction to an external attack. This involves investigating the molecule’s electrical behavior prior to, during, and following the attack by a reagent species^56^. We have computed global reactivity descriptors at the same theoretical level in order to gain a better understanding of the structural characteristics and chemical reactivity of the supposed structures. According to the following equations, the simulated parameters are ionization energy (IE), electron affinity (EA), chemical potential (µ), chemical hardness (η), chemical softness (S), electronegativity (χ), electrophilicity index (ω) and neocleophilicity index (N).

Koopmans’ theorem is used to calculate the values of IE and EA. The negative of LUMO is regarded as EA, and the negative of HOMO is taken as ionization potential. The global reactivity descriptors and the formula used to determine each are listed in Table 6.

**Table 6:** Global reactivity descriptors with their equations.

Descriptors	Equation
Ionization energy	IE = − E_HOMO_
Electron affinity	EA = − E_LUMO_
Energy gap	*∆*E = E_HOMO_ − E_LUMO_
Chemical hardness	η = Energy gap/2
Chemical potential	µ = (E_HOMO_+ E_LUMO_)/2
Chemical softness	S = 1/η
Electrophilicity index	ω = µ^2^/2η
Nucleophilicity index	*N* = 1/ω
Electronegativity	χ = − µ

As defined by Pauling^57^, electronegativity (χ) is the degree to which an atom in a molecule draws the bonded electrons towards itself, making it possibly the most important descriptor in the context of chemical reactivity.

The electronic chemical potential (µ) of a molecule indicates its electronegativity. Lower electronegativity correlates with higher molecular stability. Chemical hardness (η) is a thermodynamic indicator of a substance’s reactivity and stability. Harder molecules are more stable^60^. The electrophilicity index (ω) indicates the stability of a system after absorbing electrons. Higher ω indicates a more electrophilic molecule, while lower ω indicates a more nucleophilic molecule^61^. Table 7 shows that the interaction of ZnO and CuO with GO causes the IE, µ, η, and ω of GO to decrease while, EA, S, χ, and N increased indicating increased reactivity of GO.

The CMC’s electronic stability is indicated by the computed significant IE value of 2.013 eV, and its EA value is 1.415 eV. The computed reactivity descriptors show that CMC is the most stable (least reactive) structure, while CMC/CuO/GO is the most reactive. These findings are consistent with the TDM and ΔE results in Table 5.

The obtained value of IE for the GO model is 5.099 eV, while its EA is 3.536 eV. The calculated significant IE value reflects the electronic stability of the supposed structure of GO. It also reflects a less electropositive nature and the high energy needed to remove an electron. Additionally, the GO model is favorable for gaining extra electrons. However, the interaction of CMC with the proposed structure of CuO/GO causes the IE values to decrease to 2.505, 3.338, and 4.017 eV, while the EA values dropped to 2.505, 3.017, and 3.002 eV for the three interaction mechanisms.

**Fig. 8:**
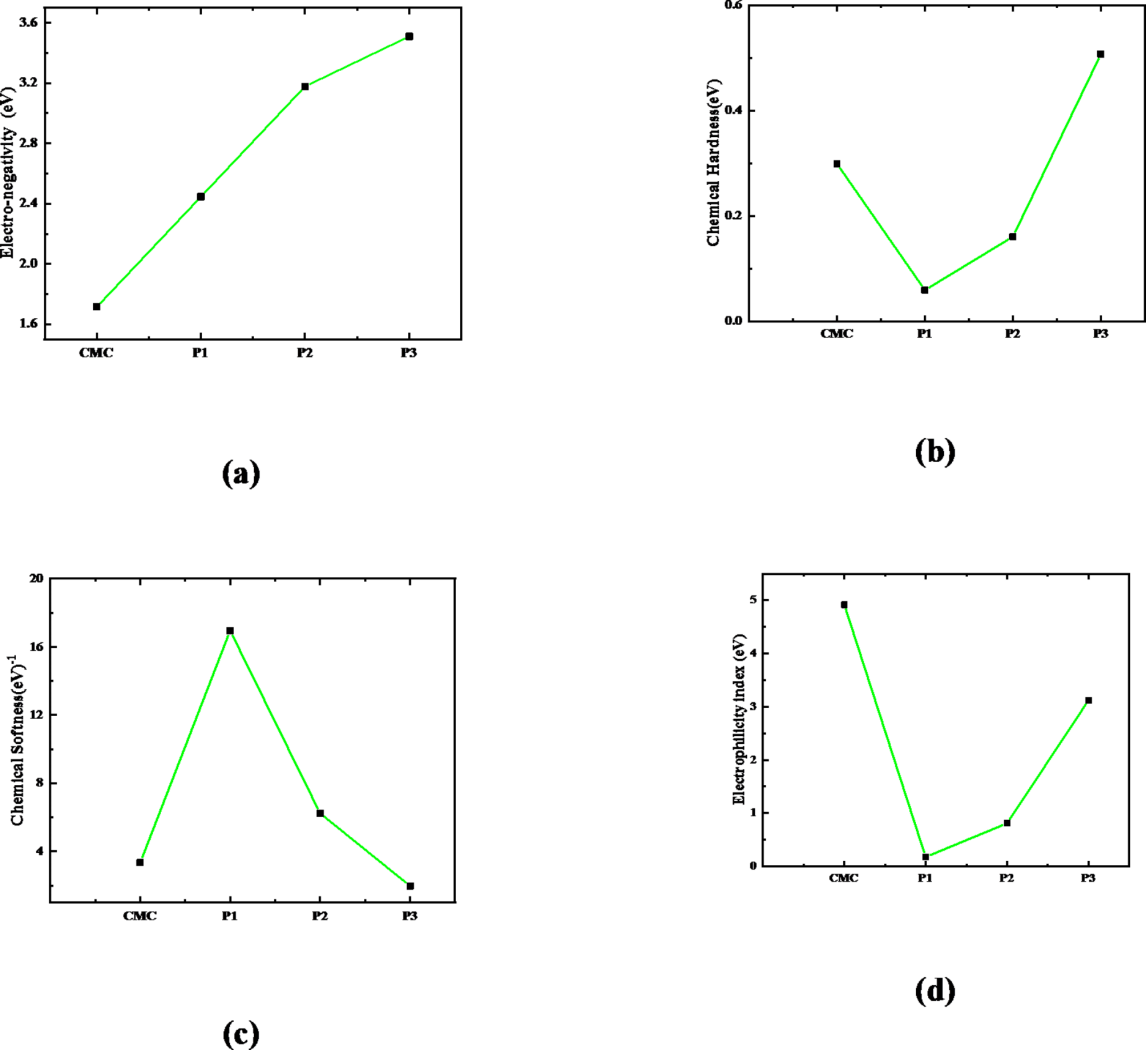
Variation of the (**a**) chemical hardness, (**b**) chemical softness, (**c**) electronegativity, and (**d**) electrophilicity index for CMC due to the interaction with CuO/GO model for the three probabilities of the interaction.

The variation in the electronegativity of CMC due to functionalization with CuO/GO model is presented in Fig. 8-a. The figure shows that the highest electronegativity value (3.510 eV) is that for the structure representing CMC interacted with CuO/GO model throughout the O atom of CuO.

The calculated electronic µ indicates an electron’s tendency to run away from an equilibrium system; similarly, the µ value is − 1.714 eV for CMC, while for CMC interacted with CuO/GO model, it decreased to − 2.446, − 3.178, − 3.510 eV for the three interaction probabilities. The observed chemical potential is more negative for the second and third interaction probabilities, indicating that these molecules are more stable because it will prevent the electron from escaping from the system.

Another metric to categorize the CMC’s softness and reactivity is its quantum chemical hardness. With a chemical hardness value of 0.299 and 0.782 eV for CMC and GO, respectively, the molecule is more stable and softer in nature. However, due to the interaction, the chemical hardness decreased for the two first probabilities of interaction but increased for the interaction proceeds through the oxygen atom of the metal oxide (see Fig. 8-b). While, the chemical softness follows the reverse reaction. As presented in Table 6; Fig. 8-c, the highest softness value belongs to the first interaction probability (interaction through the COOH group). The higher the softness value, the higher the reactivity of the supposed model molecule. Finally, the decreased values of the electrophilicity index and increased nucleophilicity index of CMC due to interaction with CuO/GO reflects he nucleophilic nature of the three interaction mechanisms (see Fig. 8-d).

These parameters indicate that the structural properties of the studied CuO/GO model molecule affect the CMC’s chemical reactivity. CMC/CuO/GO has a lower (higher) chemical hardness (softness) value than all other molecules studied. Thus, CMC/CuO/GO is discovered to be more reactive than CMC, whereas CMC is less reactive. This is also consistent with the frontier orbital energy gap, which is as follows: CMC/CuO/GO < GO/CuO < GO/ZnO < CMC, GO.

**Table 7:** B3LYP/6–31 g (d, p) calculated: ionization energy IE, electron affinity (EA), electronegativity (χ), chemical potential (µ), chemical hardness (η), chemical softness (S), electrophilicity index (ω), and nucleophilicity index (N) for CMC, GO, ZnO/GO, CuO/GO, and CMC/CuO/GO model molecules.

Structure	IE(eV)	EA (eV)	χ (eV)	µ (eV)	η (eV)	S (eV)^−1^	ω (eV)	(*N*) (eV)^−1^
CMC	2.013	1.415	1.714	− 1.714	0.299	3.347	4.917	0.203
GO	5.099	3.536	4.318	− 4.318	0.782	1.280	7.286	0.137
GO (OH)-ZnO	5.056	4.668	4.862	− 4.862	0.194	5.147	2.296	0.435
GO -(COOH)-ZnO	5.032	4.471	4.751	− 4.751	0.280	3.566	3.165	0.316
GO (OH)-CuO	5.078	4.584	4.831	− 4.831	0.248	4.052	2.880	0.347
GO-(COOH)- CuO	4.985	4.611	4.798	− 4.798	0.187	5.341	2.155	0.464
CMC-(COOH)CuO-GO (P1)	2.505	2.387	2.446	− 2.446	0.059	16.949	0.176	5.666
CMC-(OH) CuO - GO (P2)	3.338	3.017	3.178	− 3.178	0.161	6.231	0.810	1.234
CMC- (OCu) CuO- GO (P3)	4.017	3.002	3.510	− 3.510	0.507	1.970	3.125	0.320

#### Molecular electrostatic potential (MESP)

**Fig. 9:**
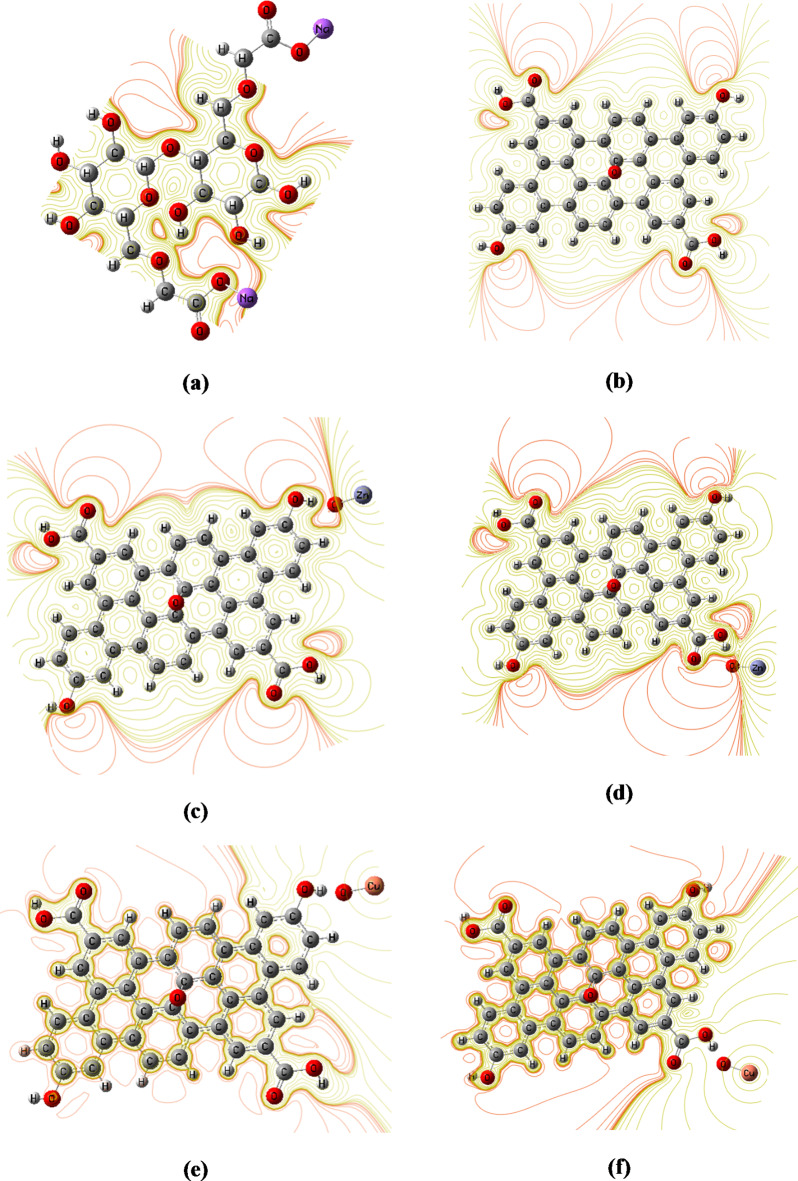

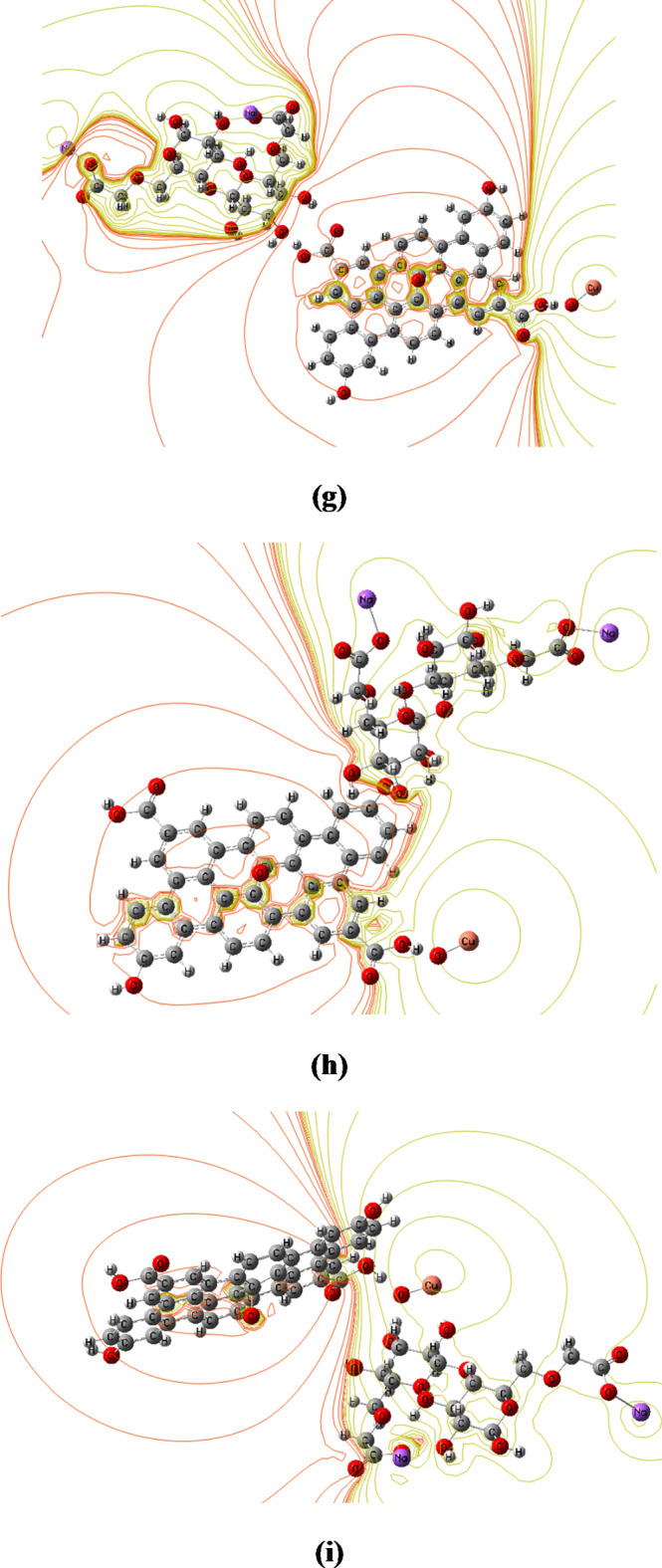
B3LYP/LanL2DZ calculated MESP maps of (**a**) CMC, (**b**) GO, (**c**) and (**d**) GO interacted with ZnO through COOH and OH functional groups, respectively, (**e**) and (**f**) GO interacted with CuO through COOH and OH functional groups, respectively, and (**g**), (**h**), and (**i**) the three probable interactions of CMC/CuO/GO model molecules.

The MESP can be used to calculate the electrophilic (electron-rich region) and nucleophilic (electron-poor region) reactive sites. The red and blue regions of the MESP represent electron abundant and electron poor areas, respectively, whereas the green area represents a nearly neutral region. Anticipating opposing areas of electrostatic potential at the binding site, a molecule’s variation in electrostatic potential largely drives medicine’s binding to receptor binding sites. Figure 9 depicts a MESP map of all the proposed structures generated at the same theoretical level using Gauss View software. The potential decreases in the following order: red > orange > yellow > green > blue^13,19,62^.

For CMC and GO model molecules, a region around the oxygen atoms in the MESP map (Fig. 9-a and b) is colored red, indicating a negative potential (electrophilic attack). Around the hydrogen atoms, a region is colored green, indicating a neutral electrostatic potential.

The MESP map shows that, in CMC and GO, the carbon atoms are in neutral potential regions (green). Meanwhile, for GO interacted with ZnO through the two mechanisms of interaction, Fig. 9-c and d showed that the electronic charges are redistributed within the GO structure and that the electronegativity increased as the red regions increased. Additionally, the figure showed that the reactivity of GO extended to its terminals, but the middle of the structure remains neutral (green regions around carbon atoms). However, due to the interaction of GO with CuO, the whole structure of GO becomes more reactive. The orange-red regions in Fig. 9-e and f show a negative electrostatic potential over the whole structure of GO with increased intensity compared to the ZnO/GO models. The orange-red regions in Fig. 9-e and f reflect the binding site for electrophilic attack. Figure 9-g, h, and i show the change in color and charge distribution of CMC after interaction with CuO/GO nanocomposite through the COOH group, OH group, and O atom of CuO, respectively. The increased red regions (low potential) indicate high reactivity and the most likely interaction site, which is susceptible to electrophile attack. Furthermore, the figures demonstrated that red-orange regions extended to the CMC and GO structures in the first probability of interaction, confirming the increased reactivity of CMC. While the interaction proceeds through the OH group and oxygen atom of CuO, the red color located only around the GO and the CMC becomes neutral. Furthermore, MESP results are consistent with those of χ, ω, and N. Additionally, MESP maps confirms the SEM results, which reflect the increased reactivity of CMC’s surface due to functionalization with CuO/GO nanocomposite.

### Antibacterial activity

**Fig. 10:**
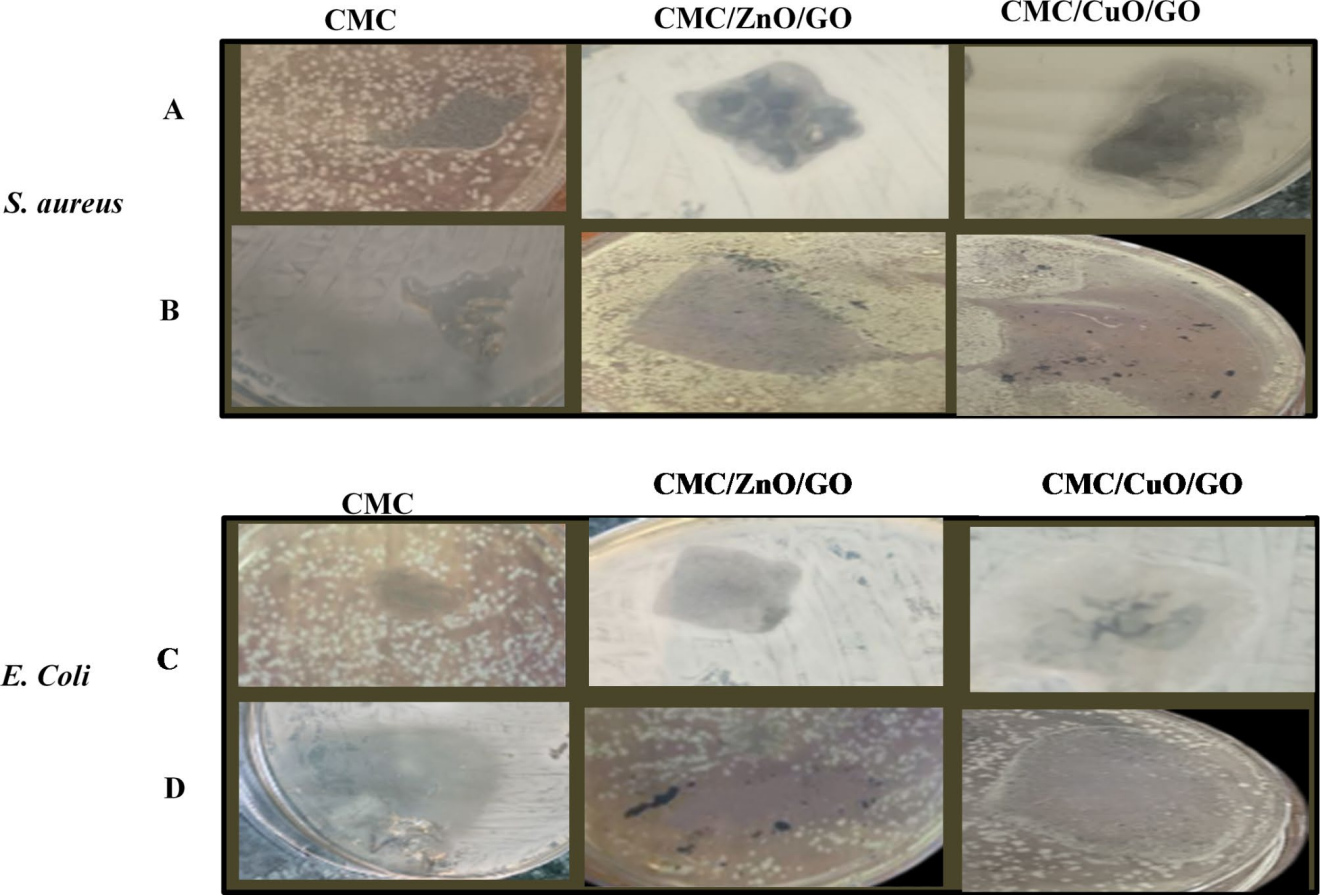
Comparison of inhibition zone assay of different nanocomposite films against *Staphylococcus aureus* and *Escherichia coli.***A**,** C**—without laser exposure, **B**, **D**—with laser exposure.

The antibacterial properties of the pure CMC, CMC-ZnO-GO and CMC-CuO -GO were tested against *S. aureus* and *E. coli*. The inhibition zone data, presented in Table 8; Fig. 10, show that both CMC/ZnO/GO and CMC/CuO/GO demonstrated antibacterial activity higher than CMC alone, particularly against *S. aureus*, due to the presence of ZnO and CuO NPs. The inhibition zones for CMC/ZnO/GO and CMC/CuO/GO nanocomposite films against *S. aureus* were 16 mm and 14 mm, respectively. This suggests that *S. aureus* is more vulnerable to these materials compared to *E. coli*.

The Zone of Inhibition (ZOI) values for the synthesized ZnO/GO and CuO/GO nanocomposites indicate their potential effectiveness against bacterial pathogens. For ZnO/GO, the ZOI values are expected to be 12–18 mm against *S. aureus* and 10–16 mm against *E. coli*^63^. In contrast, CuO/GO nanocomposites are anticipated to exhibit ZOI values of 14–22 mm for *S. aureus* and 12–20 mm for *E. coli*^64^. These values align with our experimental results, which demonstrate significant antibacterial activity for both nanocomposites. The observed ZOI values reflect greater membrane disruption and inhibition of bacterial growth, consistent with literature reports on similar nanocomposite systems^65,66^. This suggests a promising application of these nanocomposites in antimicrobial coatings, wound dressings, and other biomedical applications where bacterial contamination is a critical concern. Our findings support the potential use of ZnO/GO and CuO/GO nanocomposites as effective antibacterial agents.

The difference in antibacterial efficacy is likely due to the structural differences in the cell walls of the bacteria. *E. coli* has two cytoplasmic membranes, making it harder for the drug to penetrate, while *S. aureus* has a thinner membrane and lacks an outer lipid layer, allowing easier drug entry. The antibacterial activity of CMC/ZnO/GO and CMC/CuO/GO nanocomposite films was significantly higher than that of CMC alone.

CMC/CuO/GO showed the highest antibacterial effects, with activity rates of 118% against *S. aureus*, and 115% against *E. coli*, respectively. In comparison, the CMC/ZnO/GO and CMC/CuO/GO nanocomposite films without laser exposure much lower activity, with rates of 72% against *S. aureus* and 70% against *E. coli*. Interestingly, CMC/ZnO/GO and CMC/CuO/GO films after exposure to laser were even more effective than amoxicillin against *S. aureus*. These results indicate that laser exposure is effectively and successfully inhibits both *E. coli* and *S. aureus*.

The values for minimum inhibitory concentration (MIC) and half maximal inhibitory concentration (IC50) based on existing literature regarding similar ZnO/GO and CuO/GO nanocomposites. The MIC values are reported as 12–18 µg/mL against *S. aureus* and 10–16 µg/mL against *E. co*li for ZnO/GO^67^, while CuO/GO exhibits MIC values of 14–22 µg/mL against *S. aureus* and 12–20 µg/mL against *E. coli*^68^. Additionally, the IC50 values for these nanocomposites are estimated to be in the range of 20–50 µg/mL for both bacterial strains. These findings underscore the potential bioactivity of the nanocomposites for antimicrobial applications, highlighting the necessity for direct measurements to validate these values in future research.

Both film thickness and surface morphology play critical roles in determining the antibacterial efficacy of our CMC/ZnO/GO and CMC/CuO/GO nanocomposites. Thicker films may limit the diffusion of reactive oxygen species (ROS) and other antibacterial agents to the surface, potentially reducing the contact with bacterial cells. In contrast, thinner films may enhance the interaction with bacteria, but their mechanical integrity could be compromised. Similarly, the surface morphology significantly influences bacterial adhesion, and rougher surfaces tend to increase contact points with bacterial cells, leading to more efficient ROS-mediated antibacterial activity^69–74^. Future work will explore how parameters such as film thickness and morphology further influence these effects.

**Fig. 11:**
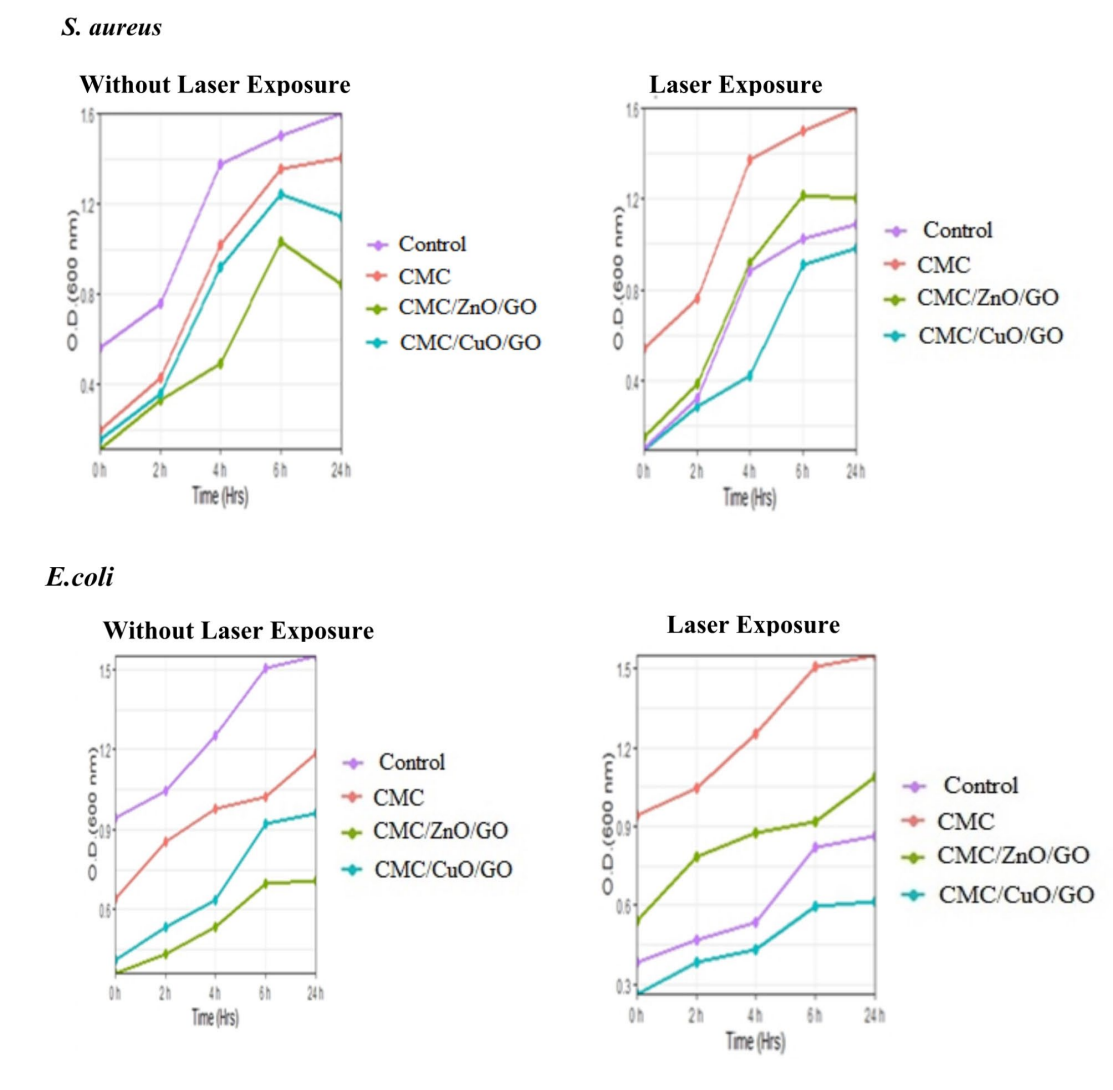
The antibacterial activity of different nanocomposite films was evaluated using a growth curve assay, measuring optical density (OD). As the different nanocomposite films exposure to laser, the OD at 600 nm decreased for each pathogen *S. aureus* and *E. coli* (represented by distinct symbols on the graph).

Figure 11 displays the laser exposure and without laser exposure growth data. The optical density (OD) values laser exposure are lower than the without laser exposure ones, the time-dependent behavior of the measured OD. Additionally, Fig. 11 shows that the contribution of nanoparticles to the OD with laser exposure is greater than that of the without laser exposure. While the bacterial concentration decreases, this reduction is on a scale comparable to the spectrophotometer’s sensitivity. It is generally recognized that nanocomposite films have antibacterial activity.

**Table 8:** The antibacterial activity of pure CMC, CMC/ZnO/GO and CMC/CuO/GO nanocomposite films.

Samples	*S. aureus*		*E. coli*	
	ZOI (mm)	% Activity Index	ZOI (mm)	% Activity Index
CMC	12	54	10	50
CMC/ZnO/GO	14	63	12	60
CMC/CuO/GO	16	72	14	70
L + CMC	20	91	16	80
L+ CMC/ZnO/GO	24	109	21	105
L+ CMC/CuO/GO	26	118	23	115
Amoxicillin^a^	22	–	20	–

^a^ The activity was measured after one-day incubation with the prepared nanocomposites. Amoxicillin was used as the reference. All experiments were done in replicates.

**Fig. 12:**
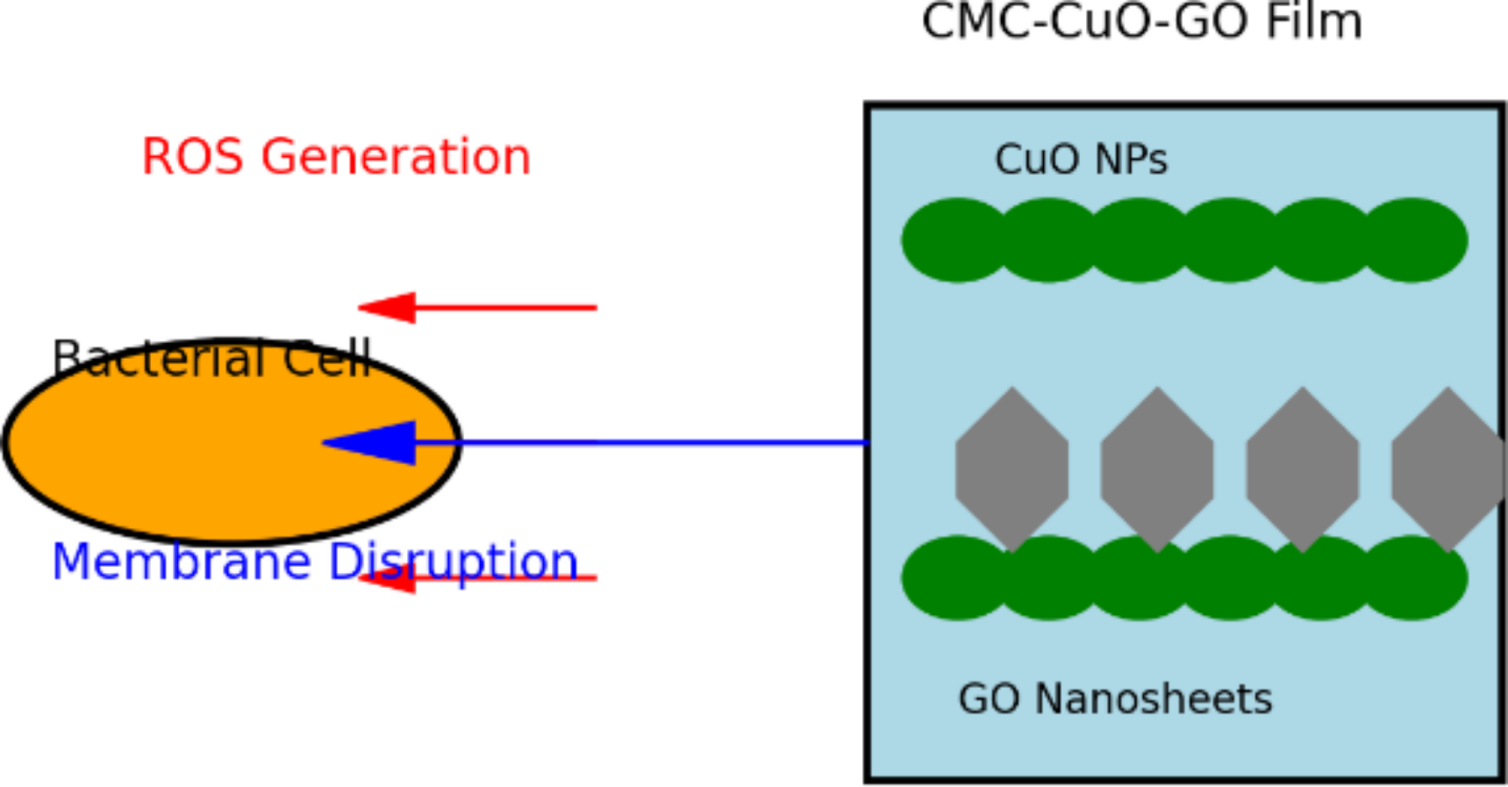
Mechanism diagram for the antibacterial activity of the CMC/CuO/GO nanocomposite film.

The antibacterial activity of the CMC/CuO/GO nanocomposite film can be attributed to the synergistic effects of its components. The combined effect of CuO NPs, GO, and CMC in the nanocomposite creates an optimized antibacterial platform. The mechanism, as illustrated in the proposed diagram (Fig. 12), demonstrates that both ROS generation and physical disruption of bacterial membranes are key factors in achieving high antibacterial efficacy. The CMC/ZnO/GO and CMC/CuO/GO nanocomposites developed in this study offer several distinct advantages over previously reported materials used for antibacterial applications. The combination of CMC with ZnO, CuO, and GO results in a significant enhancement in antibacterial performance, mainly due to better nanoparticle dispersion and stability. This synergy leads to improved bacterial inhibition, consistent with previous reports on the ability of ZnO and CuO NPs to generate reactive oxygen species (ROS) that disrupt bacterial cells^69,75^. Also, CMC’s biodegradable and biocompatible nature makes these nanocomposites suitable for use in sensitive applications, such as food packaging industry. Their biocompatibility aligns with the growing interest in environmentally friendly and safe materials for healthcare applications^13^. These attributes position CMC/ZnO/GO and CMC/CuO/GO nanocomposites as promising candidates for antibacterial applications compared to other reported materials.

## Conclusion

ZnO and CuO NPs were prepared utilizing the precipitation method while GO was prepared utilizing Hummer’s method. The ZnO and CuO NPs were blended with GO to prepare ZnO-GO and CuO-GO nanocomposites using the mechanical milling technique. Meanwhile, CMC/ZnO/GO and CMC/CuO/GO nanocomposite films with good UV shielding properties were synthesized utilizing the solution casting method. The structural properties of CMC, CMC/ZnO/GO, and CMC/CuO/GO nanocomposite films have been studied through FTIR, SEM, EDX, and XRD. The FTIR analysis shows that intra- and intermolecular hydrogen bonding is established between the nanocomposites and the CMC’s functional groups. Using the SEM, the surface morphology of ZnO/GO and CuO/GO nanocomposites was captured. It demonstrates that the CuO/GO composite has fewer agglomerated particles of smaller size and that the ZnO and CuO NPs were spherical in shape. XRD analysis confirms the hexagonal structure of the ZnO NPs and the monoclinic structure of CuO. The variation in crystallite size of CMC/ZnO/GO and CMC/CuO/GO nanocomposite has been calculated, and it is found to be 42.11 nm and 28.08 nm, respectively. The optical analysis confirmed that the UV-A, UV-B, and UV-C blocking percentages of CMC increased to 66%, 74%, and 80% when CMC is loaded with 2 wt% of ZnO/GO nanocomposite. The UV-A, UV-B, and UV-C blocking percentages of CMC loaded with 2 wt% of CuO/GO nanocomposite are 80%, 89%, and 100%, respectively. Moreover, the optical bandgap energy dramatically dropped when CMC and the nanocomposites interacted. Accordingly, it was concluded that the nanocomposite films exhibit potential for a range of applications, such as coatings for electronic devices, active ingredients in skin care products, and food packaging. Additionally, DFT study confirmed the experimental results about the formation of hydrogen bonding, decreasing the bandgap, and increasing the biological reactivity. Additionally, the results showed that the CMC nanocomposite films exhibited greater antibacterial activity against both gram positive and gram negative bacteria, which dedicated them to antibacterial food packaging applications. Accordingly, the CMC/ZnO/GO and CMC/CuO/GO nanocomposite films presented in this study not only demonstrate exceptional antibacterial properties but also offer a sustainable and versatile solution for addressing microbial resistance challenges. Further comparative studies with other materials will be beneficial to fully elucidate their advantages in practical applications.

## Data Availability

The data that support the findings of this study are available from the corresponding author upon reasonable request. Contact Medhat A. Ibrahim, Email: ma.khalek@nrc.sci.eg.
